# Plasma fatty acids and the risk of vascular disease and mortality outcomes in individuals with type 2 diabetes: results from the ADVANCE study

**DOI:** 10.1007/s00125-020-05162-z

**Published:** 2020-05-08

**Authors:** Katie Harris, Megumi Oshima, Naveed Sattar, Peter Würtz, Min Jun, Paul Welsh, Pavel Hamet, Stephen Harrap, Neil Poulter, John Chalmers, Mark Woodward

**Affiliations:** 1 The George Institute for Global Health, UNSW Sydney, Level 10, King George V Building, 83–117 Missenden Road, Camperdown, Sydney, NSW 2050 Australia; 2 Department of Nephrology and Laboratory Medicine, Kanazawa University, Ishikawa, Japan; 3 BHF Glasgow Cardiovascular Research Centre, Institute of Cardiovascular and Medical Sciences, University of Glasgow, Glasgow, UK; 4 Nightingale Health Ltd, Helsinki, Finland; 5 Centre de Recherche, Centre Hospitalier de l’Université de Montréal (CRCHUM), Montréal, QC Canada; 6 Department of Physiology, Royal Melbourne Hospital, University of Melbourne, Melbourne, VIC Australia; 7 Imperial Clinical Trials Unit, School of Public Health, Imperial College London, London, UK; 8 The George Institute for Global Health, University of Oxford, 1st Floor, Hayes House, 75 George Street, Oxford, OX1 2BQ UK; 9 Department of Epidemiology, Johns Hopkins University, Baltimore, MD USA

**Keywords:** Diabetes complications, Docosahexaenoic acid (DHA), *n*-3 fatty acids, Plasma Fatty acids, Type 2 diabetes

## Abstract

**Aims/hypothesis:**

This biomarker study aimed to quantify the association of essential and other plasma fatty acid biomarkers with macrovascular disease, microvascular disease and death in individuals with type 2 diabetes.

**Methods:**

A case-cohort study (*N* = 3576), including 654 macrovascular events, 341 microvascular events and 631 deaths during 5 years of (median) follow-up, was undertaken as a secondary analysis of the Action in Diabetes and Vascular Disease: Preterax and Diamicron Modified-Release Controlled Evaluation (ADVANCE) study (full details of the study design and primary endpoints of the ADVANCE trial and its case-cohort have been published previously). This current study considers new data: fatty acids measured from baseline plasma samples by proton NMR analysis. The fatty acids measured were *n*-3, docosahexaenoic acid (DHA), *n*-6, linoleic acid, and polyunsaturated, monounsaturated and saturated fatty acids. HRs were modelled per SD higher (percentage) fatty acid. C statistics and continuous net reclassification improvement were used to test the added value of fatty acids compared with traditional cardiovascular risk factors.

**Results:**

After adjustment for traditional cardiovascular risk factors, an inverse association was observed for *n*-3 fatty acids and DHA with the risk of macrovascular events (HR [95% CI]: 0.87 [0.80, 0.95] and 0.88 [0.81, 0.96], respectively, per 1 SD higher percentage), and for *n*-3 fatty acids with the risk of death (HR 0.91 [95% CI 0.84, 0.99] per 1 SD higher percentage). Such associations were also evident when investigating absolute levels of fatty acids. There were no statistically significant associations between any fatty acids and microvascular disease after adjustment. However, there was limited improvement in the predictive ability of models when any fatty acid was added.

**Conclusions/interpretation:**

Plasma *n*-3 fatty acids and DHA were found to be inversely associated with macrovascular disease, while *n*-3 fatty acids were also inversely associated with death. These results support the cardioprotective effects of *n*-3 fatty acids and DHA and further merit testing the role of high-dose supplementation with *n*-3 fatty acids in individuals with type 2 diabetes.

**Trial registration:**

ClinicalTrials.gov NCT00145925.

**Figure:**
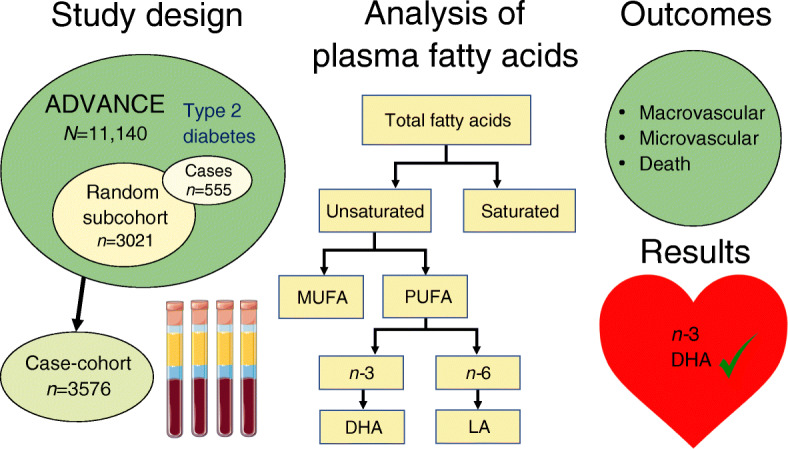
Graphical abstract

**Electronic supplementary material:**

The online version of this article (10.1007/s00125-020-05162-z) contains peer-reviewed but unedited supplementary material, which is available to authorized users.

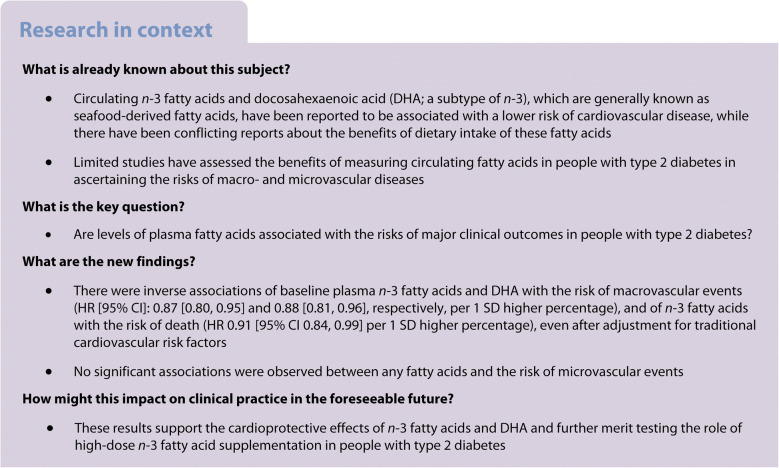



## Introduction

Type 2 diabetes is associated with a substantial risk of macrovascular disease, including coronary and cerebrovascular diseases; microvascular disease, including kidney disease and retinopathy; and premature death [1]. Early recognition of diabetes in its progression and initiation of an intervention are therefore needed for preventing such adverse long-term outcomes.

Fatty acids are vital nutrients which play regulatory roles in energy metabolism. The composition of fatty acids in blood can be affected by dietary intake [2] and has been reported to be involved in pathological mechanisms of various diseases such as insulin resistance, obesity, diabetes and atherosclerosis [3]. For several decades, clinical trials and population-based studies have attempted to determine the effects of dietary intake of fatty acids on vascular outcomes and mortality; however, the results remain controversial and inconsistent. Several meta-analyses of clinical trials have suggested that dietary intake of *n*-3 fatty acids such as docosahexaenoic acid (DHA) and eicosapentaenoic acid (EPA), which are generally known as seafood-derived fatty acids, had no, or at most a weak, protective effect on cardiovascular disease (CVD) and death in people with and without diabetes [4, 5]. In contrast, a recent meta-analysis reported that *n*-3 supplementation was associated with a lower risk of CVD [6]. In particular, the recent Reduction of Cardiovascular Events with Icosapent Ethyl–Intervention Trial (REDUCE-IT) observed pronounced protective effects of high-dose (4 g/day) supplementation with *n*-3 fatty acids against cardiovascular outcomes among individuals with established CVD or with diabetes and other risk factors [7]. Based upon these results, the recent scientific statement from the American Heart Association (AHA) recommends the prescription of *n*-3 fatty acids, whether EPA+DHA or EPA only, at a dose of 4 g/day as an effective and safe treatment for reducing triacylglycerols among individuals with hypertriglyceridaemia [8].

Blood or tissue *n*-3 fatty acids have been reported to be associated with a lower risk of CHD [9, 10]. These results support the belief that measurements of circulating *n*-3 fatty acids may be useful for the prediction and management of cardiovascular risk. However, the benefits of measuring circulating fatty acids in people with type 2 diabetes in predicting the risks of cardiovascular and other vascular diseases are unclear. In addition, most studies report the results for individual fatty acids, and there are limited studies investigating multiple circulating fatty acids together.

To address these questions, we assessed the association of baseline plasma fatty acids with the risk of macrovascular and microvascular disease and death in participants with type 2 diabetes included in the Action in Diabetes and Vascular Disease: Preterax and Diamicron Modified-Release Controlled Evaluation (ADVANCE) study.

## Methods

### Participants and study design

We performed a biomarker study using a case-cohort assessing the relationship between baseline plasma fatty acids and macrovascular and microvascular events and death in individuals with type 2 diabetes who participated in the ADVANCE trial (ClinicalTrials.gov registration no. NCT00145925). Between June 2001 and March 2003, 11,140 participants with type 2 diabetes were recruited for the ADVANCE trial, from 215 collaborating centres in 20 countries in Asia, Australasia, Europe and North America.

Individuals were potentially eligible for the ADVANCE trial if they had been diagnosed with type 2 diabetes mellitus at the age of 30 years or older and were aged 55 years or older at entry to the study. Potentially eligible participants also needed to have at least one of the following: a history of major CVD (stroke, myocardial infarction, hospital admission for transient ischaemic attack, hospital admission for unstable angina, coronary revascularisation, peripheral revascularisation, or amputation secondary to vascular disease) or at least one other risk factor for CVD. Such risk factors were defined by the presence of at least one of the following: a history of major microvascular disease (macroalbuminuria [urinary albumin/creatinine ratio >300 μg/mg], proliferative diabetic retinopathy, retinal photocoagulation therapy, macular oedema, or blindness in one eye thought to be caused by diabetes), current cigarette smoking, total cholesterol >6.0 mmol/l, HDL-cholesterol <1.0 mmol/l, microalbuminuria (urinary albumin/creatinine ratio 30–300 μg/mg), diagnosis of type 2 diabetes mellitus made ≥10 years before entry, or age ≥ 65 years at entry. The trial included two randomised interventions: (1) a double-blind assessment of the efficacy of perindopril/indapamide (2 mg/0.625 mg for 3 months, increasing to 4 mg/1.25 mg if tolerated) vs placebo; and (2) an open-label evaluation of an intensive glucose-lowering regimen using modified-release gliclazide (with a target HbA_1c_ ≤ 48 mmol/mol [6.5%]) vs standard care.

**Fig. 1:**
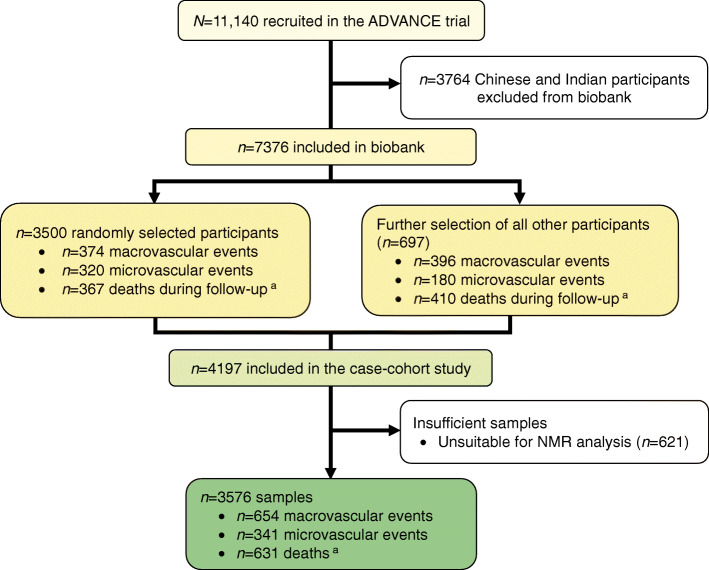
Flow diagram for design of ADVANCE case-cohort study of fatty acid biomarkers for macrovascular events, microvascular events and death. ^a^Macrovascular events, microvascular events and death are not mutually exclusive

For the present biomarker study, blood samples were available from all countries participating in the ADVANCE trial, except China and India, giving a base population of 7376 (Fig. 1). We included 4197 individuals from the case-cohort study, who comprised a random subcohort of 3500 individuals (2860 ‘controls’ and 640 ‘cases’) that was enriched with 697 additional ‘cases’ with a macrovascular or microvascular event or who had died during follow-up but were not in the subcohort. Full details of the study design and primary endpoints of the ADVANCE trial [11, 12] and its case-cohort [13] have been published elsewhere.

### Proton NMR analysis

Plasma samples were obtained at baseline from all study participants when they were in an unfasted state, given that these were people with type 2 diabetes at risk of hypoglycaemic episodes. Samples were collected across sites in a pragmatic fashion (commensurate with a multinational RCT) according to local facilities. Plasma samples were separated and stored centrally at −80°C until measurement. The present study used a previously unthawed aliquot of plasma for proton NMR (^1^H-NMR) analysis. ^1^H-NMR spectroscopy was performed on all available EDTA plasma samples from the ADVANCE case-cohort study at baseline using a low-volume (100 μl) variation of the quantitative ^1^H-NMR method (Nightingale Health, Helsinki, Finland) described previously [14, 15] and reviewed [16]. Sample spectra were analysed on a Bruker AVANCE III HD spectrometer (Billerica, MA, USA) to quantify a targeted list of metabolites, lipids and lipoproteins, as described previously [16].

This study presents new data on all fatty acids that can be robustly quantify by NMR, i.e. two individual fatty acids: DHA (an *n*-3 fatty acid) and linoleic acid (LA; an *n*-6 fatty acid). Six aggregate measures included: *n*-3, *n*-6, polyunsaturated (PUFA; sum of *n*-3 and *n*-6 fatty acids), monounsaturated (MUFA) and saturated (SFA) fatty acids. The percentages of total fatty acids as well as the absolute levels were used as the exposures of interest for the current analysis.

### Study outcomes

The primary outcomes for this study were major macrovascular and microvascular events and death that occurred during a median of 5 years of follow-up. An independent endpoint adjudication committee validated all these outcomes using ICD codes (electronic supplementary material [ESM] Methods). Major macrovascular events were cardiovascular death, non-fatal myocardial infarction and non-fatal stroke. Major microvascular events were a composite of new or worsening nephropathy or retinopathy, defined as any of the following: (1) development of macroalbuminuria; (2) doubling of serum creatinine level to ≥200 μmol/l; (3) the need for renal replacement therapy due to kidney disease, or death due to renal disease; (4) development of proliferative retinopathy; (5) development of macular oedema; (6) occurrence of diabetes-related blindness; (7) use of retinal photocoagulation therapy. The secondary outcomes were individual components of macrovascular and microvascular events: cardiovascular death, non-fatal stroke, non-fatal myocardial infarction and new or worsening nephropathy.

### Statistical analyses

Baseline participant characteristics were summarised according to the study outcomes (major macrovascular and microvascular events and death). Categorical data were presented as number (percentage), and continuous data according to the data distribution, mean (SD) for approximately symmetrically distributed data and median (interquartile interval [IQI]) for skewed distributions. The percentage contribution of the fatty acid biomarkers of the total fatty acid and the absolute fatty acid values were summarised as mean (SD) values for each study outcome.

Cox proportional hazards models for case-cohort data were used to model the associations between fatty acids and the study outcomes, adjusting covariates considered as traditional cardiovascular risk factors. Models estimated HR (per SD percentage higher) of the percentage contribution of total fatty acids. Two sets of models were fit for each fatty acid-outcome combination: model 1, adjusted for age, sex, region and the treatments randomly allocated in the RCT; and multiple-adjusted model 2, additionally adjusted for history of macrovascular disease, duration of diabetes, current smoking status, systolic BP, BMI, urinary albumin/creatinine ratio, eGFR (calculated using the Chronic Kidney Disease Epidemiology Collaboration [CKD-EPI] creatinine equation), HbA_1c_, HDL-cholesterol, triacylglycerols, and use of aspirin or other antiplatelet agents, statins or other lipid-lowering agents, β-blockers and ACE inhibitors or angiotensin receptor blockers. All *p* values reported are two-sided, with the 5% threshold used to determine statistical significance. Since several statistical tests are included in this article, the reader is recommended to treat marginal levels of significance with caution.

Sensitivity analyses included models estimating HRs for 1 SD higher of the absolute level of each fatty acid. For the fatty acid-outcome combinations that yielded statistically significant results in multiple-adjusted models in the main analysis, models were fitted estimating HRs in quarters of the range of values of the percentage of fatty acids.

Subgroup analyses were performed according to baseline covariates, including age (<65 years or ≥65 years), sex, region of residence (Australia, New Zealand and South East Asia, Canada, Continental Europe and Northern Europe), history of macrovascular disease, history of microvascular disease, eGFR (<60 ml min^−1^ [1.73 m]^−2^ or ≥60 ml min^−1^ [1.73 m]^−2^), triacylglycerols (<1.7 mmol/l or ≥1.7 mmol/l), and randomised treatments (BP- and glucose-lowering treatments).

The ability of fatty acids to discriminate between those who will and those who will not go on to suffer major macrovascular and microvascular events and death was estimated using C statistics accounting for censoring [17, 18]. Further, the ability of fatty acids to reclassify participants was estimated using the continuous net reclassification improvement (NRI) [19, 20]. These statistics were computed, for 5 year risk, for individuals in the random subcohort only for those fatty acids whose percentage contribution demonstrated a statistically significant association with outcomes. 95% CIs for the C statistic (and increments in it when adding fatty acids) and NRI were calculated using bootstrap methods with a normal approximation and 500 bootstrap iterations. All analyses in this study were performed using Stata/MP, version 15 (Stata Corporation, College Station, TX, USA), and R, version 3.5.3 (R Foundation for Statistical Computing, Vienna, Austria); the R code is provided in the ESM Methods.

## Results

### Participants’ characteristics

There were 3576 individuals from the case-cohort that had available data for at least one fatty acid, which comprised 3021 from the random subcohort (2507 controls and 514 cases) and 555 additional cases. For the 3576 individuals there were 654 macrovascular events, 341 microvascular events and 631 deaths during a median of 5 years of follow-up (Fig. 1).

Higher percentages of the fatty acids of total fatty acids in those free from an adverse event were observed, compared with lower percentages in those with adverse events. Significant differences were observed in the percentage of *n*-3 fatty acids and DHA for macrovascular events and death, and in PUFA, SFA, *n*-6 fatty acids and LA in microvascular events (Table 1). Similar findings were observed in the absolute fatty acid levels, for most fatty acids considered in this study, where the mean levels were significantly higher in participants who were free from major macrovascular events and alive at the end of the study. There were no significant differences in the absolute level of any fatty acids for microvascular events.

**Table 1:** Baseline characteristics of participants in the case-cohort study by macrovascular events, microvascular events and death

Characteristic	Macrovascular events		Microvascular events		Death	
	Yes	No	Yes	No	Yes	No
*N* (%)	654 (18.3)	2922 (81.7)	341 (9.5)	3235 (90.5)	631 (17.6)	2945 (82.4)
Age, years	69 (7)	66 (7)	66 (6)	67 (7)	70 (7)	66 (6)
Men, *n* (%)	450 (69)	1712 (59)	226 (66)	1936 (60)	438 (69)	1724 (59)
Region, *n* (%)						
ANZ/SEA	155 (24)	713 (24)	123 (36)	745 (23)	120 (19)	748 (25)
Canada	33 (5)	185 (6)	28 (8)	190 (6)	34 (5)	184 (6)
Continental Europe	262 (40)	1154 (39)	90 (26)	1326 (41)	264 (42)	1152 (39)
Northern Europe	204 (31)	870 (30)	100 (29)	974 (30)	213 (34)	861 (29)
Duration of diabetes, years	9.2 (7.1)	7.6 (6.3)	9.8 (6.9)	7.7 (6.4)	9.2 (7.6)	7.6 (6.2)
History of macrovascular disease, *n* (%)	323 (49)	925 (32)	118 (35)	1130 (35)	283 (45)	965 (33)
Current smoker, *n* (%)	84 (13)	390 (13)	44 (13)	430 (13)	96 (15)	378 (13)
Systolic BP, mmHg	150 (23)	146 (21)	150 (21)	147 (22)	149 (23)	147 (21)
Diastolic BP, mmHg	82 (11)	82 (11)	82 (11)	82 (11)	81 (12)	82 (11)
HbA_1c_, mmol/mol	60 (17)	57 (15)	61 (18)	57 (15)	59 (17)	57 (15)
HbA_1c_, %	7.6 (1.6)	7.4 (1.4)	7.8 (1.6)	7.4 (1.4)	7.6 (1.6)	7.4 (1.4)
eGFR, ml min^−1^ (1.73 m)^−2^	68 (18)	73 (16)	70 (19)	72 (16)	67 (18)	73 (16)
Urinary ACR, μg/mg	21 (9, 71)	13 (6, 35)	49 (14, 127)	13 (6, 34)	21 (8, 66)	13 (6, 35)
Total cholesterol, mmol/mol	5.1 (1.2)	5.2 (1.2)	5.2 (1.1)	5.1 (1.2)	5.1 (1.1)	5.2 (1.2)
HDL-cholesterol, mmol/mol	1.17 (0.31)	1.23 (0.33)	1.18 (0.31)	1.23 (0.33)	1.18 (0.31)	1.23 (0.33)
Triacylglycerols, mmol/l	1.6 (1.2, 2.3)	1.7 (1.2, 2.4)	1.8 (1.3, 2.6)	1.7 (1.2, 2.3)	1.6 (1.2 2.3)	1.7 (1.2 2.4)
Randomised BP-lowering treatment, *n* (%)	310 (47)	1453 (50)	163 (48)	1600 (49)	296 (47)	1467 (50)
Randomised intensive blood glucose control, *n* (%)	321 (49)	1445 (49)	151 (44)	1615 (50)	309 (49)	1457 (49)
Medication use, *n* (%)						
Aspirin or other antiplatelet agent	386 (59)	1373 (47)	170 (50)	1589 (49)	351 (56)	1408 (48)
Statins or other lipid-lowering agent	283 (43)	1305 (45)	157 (46)	1431 (44)	260 (41)	1328 (45)
β-blocker	211 (32)	875 (30)	95 (28)	991 (31)	196 (31)	890 (30)
ACE inhibitor or angiotensin receptor blocker	417 (64)	1664 (57)	231 (68)	1850 (57)	394 (62)	1687 (57)
Fatty acids, % of total fatty acids						
PUFA	28.8 (5.8)	29.0 (5.7)	28.2 (5.8)	29.1 (5.7)	28.9 (5.5)	29.0 (5.8)
*n*-3 Fatty acids	2.5 (1.3)	2.8 (1.4)	2.7 (1.4)	2.7 (1.4)	2.6 (1.3)	2.8 (1.4)
DHA	0.75 (0.46)	0.83 (0.50)	0.79 (0.53)	0.82 (0.49)	0.76 (0.48)	0.83 (0.50)
*n*-6 Fatty acids	26.2 (4.9)	26.2 (4.8)	25.5 (5.2)	26.3 (4.8)	26.3 (4.6)	26.2 (4.9)
LA	17.2 (6.7)	17.2 (6.2)	16.6 (6.7)	17.3 (6.3)	17.3 (6.3)	17.2 (6.3)
MUFA	30.2 (3.6)	30.2 (3.7)	30.4 (5.4)	30.2 (3.5)	30.2 (3.7)	30.2 (3.7)
SFA	41.0 (5.1)	40.8 (5.0)	41.6 (5.9)	40.8 (5.0)	40.9 (5.0)	40.8 (5.1)
Fatty acids, mmol/l						
Total fatty acids	8.34 (2.91)	8.63 (3.18)	8.50 (2.93)	8.59 (3.16)	8.31 (2.61)	8.64 (3.24)
PUFA	2.45 (1.01)	2.55 (1.03)	2.45 (1.03)	2.53 (1.03)	2.43 (0.92)	2.55 (1.05)
*n*-3 Fatty acids	0.23 (0.15)	0.26 (0.17)	0.24 (0.17)	0.25 (0.17)	0.23 (0.15)	0.26 (0.17)
DHA	0.067 (0.049)	0.077 (0.056)	0.073 (0.057)	0.076 (0.055)	0.068 (0.050)	0.077 (0.056)
*n*-6 Fatty acids	2.21 (0.88)	2.29 (0.89)	2.21 (0.89)	2.28 (0.88)	2.20 (0.79)	2.29 (0.90)
LA	1.52 (0.84)	1.56 (0.83)	1.50 (0.83)	1.56 (0.83)	1.51 (0.75)	1.57 (0.84)
MUFA	2.55 (1.05)	2.64 (1.16)	2.61 (1.07)	3.43 (1.15)	2.54 (0.95)	2.64 (1.17)
SFA	3.34 (1.06)	3.46 (1.23)	3.45 (1.08)	3.43 (1.22)	3.33 (0.95)	3.46 (1.25)

Data are presented as mean (SD) or median with IQI (lower quartile, upper quartile), unless otherwise stated

ACR, albumin/creatinine ratio; ANZ/SEA, Australia and New Zealand/South-East Asia; IQI, interquartile interval

### Clinical outcomes during follow-up

**Fig. 2:**
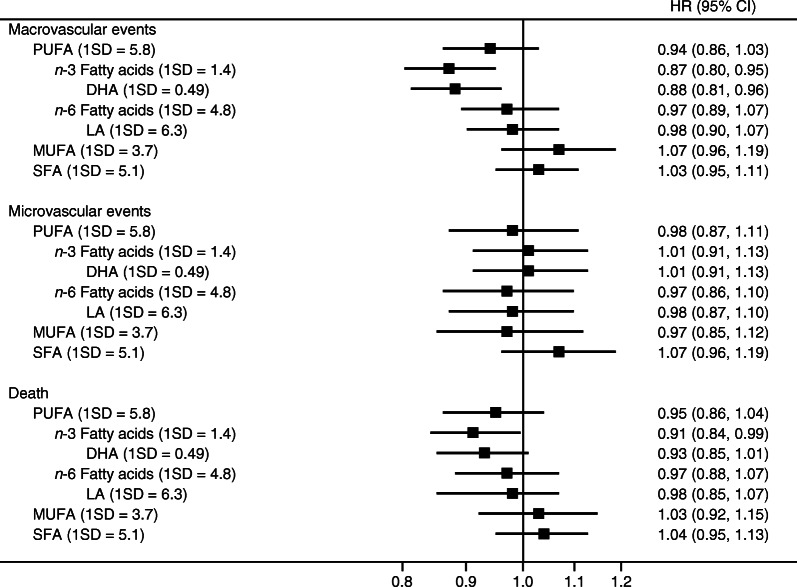
Adjusted HRs for macrovascular events, microvascular events and death associated with fatty acid levels (per 1 SD increase in percentage of total fatty acids), using multiple-adjusted models. Models were adjusted for age, sex, region, randomised treatment, history of macrovascular disease, duration of diabetes, current smoking status, systolic BP, BMI, urinary albumin/creatinine ratio, eGFR, HbA_1c_, HDL-cholesterol, triacylglycerols, and use of aspirin or other antiplatelet agents, statins or other lipid-lowering agents, β-blockers, and ACE inhibitors or angiotensin receptor blockers

After adjustment for age, sex, region and randomised treatments (model 1), there were highly significant inverse associations with the risk of macrovascular events and death for the percentage of *n*-3 fatty acids (HR [95% CI]: 0.84 [0.77, 0.91] and 0.85 [0.79, 0.93], respectively, per 1 SD higher percentage) and DHA (HR [95% CI]: 0.82 [0.76, 0.89]) and 0.85 [0.78, 0.92], respectively, per 1 SD higher percentage) (ESM Fig. 1). After further adjustment (model 2), the association remained, albeit weaker, for the percentage of *n*-3 fatty acids (HR [95% CI]: 0.87 [0.80, 0.95] and 0.91 [0.84, 0.99] for the risk of macrovascular events and death, respectively, per 1 SD higher percentage) and for DHA (HR [95% CI]: 0.88 [0.81, 0.96] and 0.93 [0.85, 1.01] for the risk of macrovascular events and death, respectively, per 1 SD higher percentage), although the latter was not significant (Fig. 2).

**Fig. 3:**
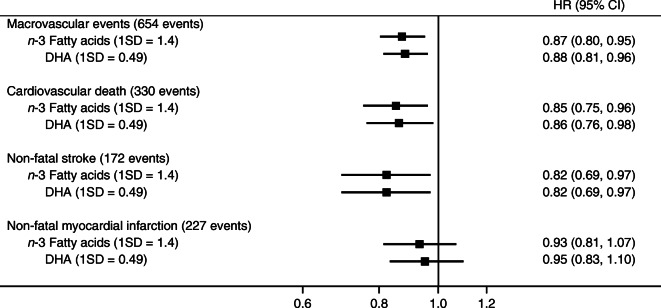
Adjusted HRs for individual components of macrovascular events (cardiovascular death, non-fatal myocardial infarction, non-fatal stroke) associated with *n*-3 fatty acid and DHA levels (per 1 SD increase in percentage of total fatty acids,) using multiple-adjusted models. Models were adjusted for age, sex, region, randomised treatment, history of macrovascular disease, duration of diabetes, current smoking status, systolic BP, BMI, urinary albumin/creatinine ratio, eGFR, Hb_1c_, HDL-cholesterol, triacylglycerols, and use of aspirin or other antiplatelet agents, statins or other lipid-lowering agents, β-blockers, ACE inhibitors or angiotensin receptor blockers

For individual components of macrovascular events from multiple-adjusted models, *n*-3 fatty acids and DHA were associated with lower risks of cardiovascular death (HR [95%CI]: 0.85 [0.75, 0.96] and 0.86 [0.76, 0.98]), respectively, and non-fatal stroke (HR [95%CI]: 0.82 [0.69, 0.97] and 0.82 [0.69, 0.97]), respectively (Fig. 3). The statistically significant associations did not hold for non-fatal myocardial infarction.

Further, significant inverse associations were suggested after minor adjustment in the percentage of PUFA on macrovascular events, and in PUFA, *n*-6 fatty acids and LA on the risk of death (ESM Fig. 1), but these findings did not persist after further adjustment (Fig. 2). No significant associations were observed between any fatty acids and the risk of microvascular events (Fig. 2) as well as new or worsening nephropathy (ESM Fig. 2).

Further analyses of quarters of fatty acids levels revealed that the inverse associations with the percentage of *n*-3 and DHA were approximately linear with the adjusted HRs for macrovascular events and death (ESM Fig. 3). The associations in the absolute values of the fatty acids were in line with the percentage fatty acid, albeit weaker (ESM Fig. 4).

### Subgroup analyses for *n*-3 fatty acids and DHA

Subgroup analyses were undertaken for *n*-3 fatty acids and DHA with macrovascular events and death (ESM Figs 5 and 6). There were no significant interactions between any subgroups (*p* for interaction >0.1) other than triacylglycerols (*p* for interaction = 0.02) and randomised BP-lowering treatment (*p* for interaction = 0.01) for *n*-3 fatty acids and the risk of macrovascular events. A further subgroup analysis (ESM Table 1) for major microvascular outcomes in those free from microvascular disease at baseline yielded HRs [95% CI] for *n*-3 of 0.94 [0.82, 1.08] and for DHA of 0.94 [0.82, 1.08]. The *p* value for interaction indicates that there was no statistically significant interaction in the association of *n*-3 (*p* = 0.243) or DHA (*p* = 0.247) and major microvascular outcomes by history of microvascular disease at baseline.

### Prognostic value of fatty acids compared with traditional risk factors

The difference in the C statistics between the base model with age, sex, region and randomised treatments without fatty acids and the model which included the index fatty acids demonstrated small improvements for predicting macrovascular events for DHA (difference: 0.0104 [95% CI 0.0001, 0.0206]) and for predicting death for *n*-3 fatty acids (difference: 0.0103 [95% CI 0.0004, 0.0202]) and DHA (difference: 0.0084 [95% CI 0.0000, 0.0169]) (ESM Table 2). There were no statistically significant differences in the C statistics for other fatty acids. The inclusion of *n*-3 fatty acids and DHA, in comparison with base model 1, yielded the largest improvements in the continuous NRI, although not statistically significant: 0.157 (95% CI −0.006, 0.267) and 0.156 (95% CI −0.009, 0.265), respectively, for macrovascular events; and 0.170 (95% CI −0.013, 0.293) and 0.161 (95% CI −0.008, 0.272), respectively, for death (ESM Table 2). After including fatty acids in model 2, which included many traditional cardiovascular risk factors, there were limited improvements in the C statistic and continuous NRI for *n*-3 fatty acids and DHA, although there were no longer any significant associations (Table 2).

**Table 2:** Prognostic value of fatty acids, compared with traditional CVD risk factors, using C statistic (and difference) and continuous NRI, with 95% CIs, for macrovascular events and death (per 1 SD increase in percentage of total fatty acids): results from multiple-adjusted models

	C statistic and difference (95% CI)^a^	Continuous NRI (95% CI)
Macrovascular events		
Multiple-adjusted model 2^b^	0.6919 (0.6467, 0.7101)	–
+ *n*-3 Fatty acids	+0.0034 (−0.0035, 0.0102)	0.144 (−0.019, 0.263)
+ DHA	+0.0025 (−0.0027, 0.0076)	0.121 (−0.066, 0.254)
Death		
Multiple-adjusted model 2^b^	0.7044 (0.6641, 0.7220)	–
+ *n*-3 Fatty acids	+0.0057 (−0.0005, 0.0119)	0.124 (−0.047, 0.257)
+ DHA	+0.0042 (−0.0004, 0.0087)	0.145 (−0.070, 0.274)

^a^Presented for the multiple-adjusted model with traditional CVD risk factors; the difference in the C statistic given the addition of each fatty acid is presented as a difference with 95% CI of the difference

^b^Adjusted for age, sex, region, randomised treatment, history of macrovascular disease, duration of diabetes, current smoking status, systolic BP, BMI, urinary albumin/creatinine ratio, eGFR, HbA_1c_, HDL-cholesterol, triacylglycerols, and use of aspirin or other antiplatelet agents, statins or other lipid-lowering agents, β-blockers, and ACE inhibitors or angiotensin receptor blockers

## Discussion

This biomarker study showed inverse associations of baseline plasma *n*-3 fatty acids and DHA with the risk of macrovascular events and for *n*-3 fatty acids with the risk of death among individuals with type 2 diabetes. These inverse associations appeared approximately linear, and among the macrovascular events, *n*-3 fatty acids and DHA demonstrated stronger associations with cardiovascular death and non-fatal stroke. In contrast, no significant associations were observed for the predominant fatty acids such as *n*-6 fatty acids, LA, MUFA and SFA with the risk of macrovascular events and death, after adjustment for multiple traditional risk factors.

The prospective associations of circulating *n*-3 fatty acids and DHA with the risk of CVD are consistent with the prior studies. According to previous pooled analyses, DHA in whole plasma was associated with a lower risk of fatal and non-fatal CHD (RR [95% CI]: 0.78 [0.69, 0.90] and 0.91 [0.84, 0.98], respectively) in people without a history of CVD [9]. In addition, another study reported that plasma *n*-3 fatty acids were associated with a lower risk of non-fatal myocardial infarction [21]. Similar inverse associations with CHD and stroke were observed in other studies which measured *n*-3 fatty acids in plasma phospholipid [10, 22–24], whole blood [25] and serum [26], among people without prior CVD. These studies did not assess the C statistic and NRI; however, the present study did not detect significant improvement in predicting macrovascular events by adding *n*-3 fatty acids or DHA into the model including traditional cardiovascular risk factors. This indicates that the predictive power of plasma *n*-3 fatty acids and DHA may be limited in the presence of a complement of traditional cardiovascular risk factors. On the other hand, cardiovascular benefits of high-dose supplementation with *n*-3 fatty acids were recently observed in REDUCE-IT, where there were potentially greater benefits of *n*-3 fatty acid supplementation in those with lower plasma levels of *n*-3 fatty acids.

In contrast to generally consistent results from observational studies of circulating *n*-3 fatty acids and DHA, the effects of *n*-3 fatty acid supplementation on cardiovascular outcomes in RCTs have been mixed [4, 6, 27–30]. The recent study, A Study of Cardiovascular Events in Diabetes (ASCEND) trial, of 15,480 individuals with diabetes free of prior CVD, which tested *n*-3 fatty acid supplementation (1 g/day) for 7.4 years, did not lower the risk of composite major vascular outcomes, while only vascular deaths were less frequent in the supplementation group than in the placebo group (RR 0.82 [95% CI 0.68, 0.98]) [31]. On the other hand, the recent AHA science advisory has suggested that the use of *n*-3 fatty acid supplementation was probably justified in individuals at high cardiovascular risk [8, 28]. In REDUCE-IT, which used high-dose (4 g/day) *n*-3 fatty acid supplementation in 8179 individuals with established CVD or with diabetes and other risk factors, the risk of composite cardiovascular outcomes was substantially reduced (HR 0.75 [95% CI 0.68, 0.83]) [7]. In addition, some secondary analyses from large trials have reported the benefit of *n*-3 fatty acid supplementation on CVD in diabetic populations with hypercholesterolaemia [32], chronic heart failure [33] and history of myocardial infarction [34]. Further investigation of the benefits of *n*-3 fatty acid supplementation will, therefore, be required among people with type 2 diabetes and high CVD risks.

Several mechanisms may explain the favourable associations between *n*-3 fatty acids and the risk of CVD. Previous clinical trials looking at intermediate cardiovascular outcomes among people with diabetes have reported that *n*-3 fatty acid supplementation could lower triacylglycerol concentrations [35, 36], improve arterial blood flow and attenuate inflammatory signals [37, 38]. These effects were supported by clinical trials in the general population and in experimental studies [39–41].

The present study observed the inverse associations between baseline plasma *n*-6 fatty acids and LA with the risk of death, but these associations attenuated after adjustment for multiple risk factors. Similar findings have been reported between serum LA and mortality in a cohort of older adults (≥65 years of age) [42]. We did not, however, detect significant associations between *n*-6 fatty acids and LA with the risk of macrovascular events. These results were consistent with those of previous observational studies [23, 24, 43] and directionally concordant with the recent pooled analyses of 30 cohort studies which reported that higher circulating and tissue levels of LA were associated with a lower risk of major cardiovascular events (HR 0.93 [95% CI 0.88, 0.99]) [44]. In addition, our study did not detect significant associations with the risk of CVD for circulating MUFA and SFA, which is consistent with previous studies [24, 43, 45], while some studies have reported that MUFA in blood were associated with a higher risk of CVD [46].

There were no significant associations between any fatty acids and the risk of microvascular events. A further analysis specifically for renal outcome also demonstrated no significant associations. Although limited studies have assessed the association between circulating fatty acids and renal outcomes, in an Italian cohort of 931 adults, plasma *n*-3 fatty acids were inversely associated with the risk of developing renal insufficiency (creatinine clearance rate <60 ml/min) [47]. However, in a cohort of 2792 individuals, levels of *n*-3 and *n*-6 fatty acids and SFA in plasma phospholipid were not associated with kidney function [48].

The strengths of the current study include the use of an efficiently designed case-cohort study from a well-characterised clinical trial to yield a powerful study for a range of outcomes, which were independently adjudicated according to pre-specified criteria. This study included multiple plasma fatty acids and the ability to adjust for multiple covariates including lipids and lipid-lowering drugs, such as statins. We also considered the percentage that individual fatty acids contributed to total fatty acids, as well as the absolute levels of fatty acid, and both measures are important for interpreting fatty acid values since an increased intake of a specific fatty acid could alter the relative percentage of other fatty acids while their absolute levels are unlikely to be altered. The present study, however, has several limitations. First, as the study cohort was derived from a randomised trial of individuals with type 2 diabetes, our results may have limited generalisability to broader populations. Second, as fatty acids were measured in pragmatically collected plasma samples in a randomised trial, we cannot rule out the potential for differential pre-analytical sample handling or sample degradation during storage, which may have biased our results [49]. Further, as plasma samples were collected from non-fasted participants, the levels of fatty acids might have been affected by the consumption of a recent meal [50], although, in clinical practice, fasting is rarely required among individuals with type 2 diabetes. Third, fatty acids were only measured in plasma samples collected at study baseline; thus, we were unable to consider how the change in fatty acid values during the study follow-up might have influenced the exposure–outcome association. Finally, our study considered only two individual fatty acids (DHA and LA), since the resolution of the employed high-throughput NMR platform was limited in terms of individual fatty acid types, and only allowed robust quantification of DHA within *n*-3, and LA within *n*-6. The specific set of measures was determined by their overall concentration in plasma and also on spectroscopic aspects, such as overlapping signals, which makes it challenging to quantify from native plasma where no lipid extraction is used [14]. LA and DHA were reported since they generate distinct peaks in the spectral data produced by the measurement, and we were able quantify them separately as part of our high-throughput service. However, fatty acid concentrations quantified by the NMR metabolomics platform were highly consistent with the concentrations compared with GC, the latter being challenging with large samples [46]. Further, NMR, is a novel technology with the potential of offering a cost-effective platform for multiple biomarker testing and has great potential in regard to fatty acid measurement.

In conclusion, we report distinct associations of different plasma fatty acids with the risk of major clinical outcomes in individuals with type 2 diabetes. In particular, plasma *n*-3 fatty acids were associated with a lower risk of macrovascular disease and death, and DHA was associated with a lower risk of macrovascular disease. These results support the cardioprotective effects of *n*-3 fatty acids and DHA and further merit testing the role of high-dose *n*-3 fatty acid supplementation in individuals with type 2 diabetes.

## Electronic supplementary material

ESM (PDF 2.13 mb)

## Data Availability

Restrictions apply to the availability of these data, which were used by agreement of the ADVANCE steering committee for the current study, and so are not publicly available.

## Abbreviations

ADVANCE: Action in Diabetes and Vascular Disease: Preterax and Diamicron Modified-Release Controlled Evaluation
AHA: American Heart Association
CVD: Cardiovascular disease
DHA: Docosahexaenoic acid
EPA: Eicosapentaenoic acid
^1^H-NMR: Proton NMR
LA: Linoleic acid
MUFA: Monounsaturated fatty acids
NRI: Net reclassification index
PUFA: Polyunsaturated fatty acids
REDUCE-IT: Reduction of Cardiovascular Events with Icosapent Ethyl–Intervention Trial
SFA: Saturated fatty acids

## Authors’ relationships and activities

MW reports consultancy fees from Amgen and Kirin and is supported by an Australian NHMRC fellowship (APP1080206) and MRC program grant (APP1149987). JC received research grants from the NHMRC of Australia and from Servier for the ADVANCE trial and ADVANCE-ON post-trial follow-up, and honoraria for speaking about these studies at scientific meetings; he also reports grant support from a program grant (APP1149987) from the NHMRC of Australia. MJ reports receiving grant support from the NHMRC of Australia (project grant: 1148060) and unrestricted grant support from VentureWise (a wholly owned commercial subsidiary of NPS MedicineWise) to conduct a commissioned project funded by AstraZeneca. PWü is an employee and shareholder of Nightingale Health Ltd., which conducted the biomarker quantification. NP has received personal speaker fees from Servier, Takeda and Novo Nordisk, and advisory board activities from Astra Zeneca and Novo Nordisk. He has also received grants for his research group relating to type 2 diabetes mellitus from Diabetes UK, NIHR EME, Julius Clinical and the British Heart Foundation. He holds no stocks and shares in any such company. All other authors declare that there is no duality of interest associated with their contribution to this manuscript.
