# Supplementary material for: Plasma fatty acids and the risk of vascular disease and mortality outcomes in individuals with type 2 diabetes: results from the ADVANCE study
Source: Diabetologia. 2020 May 8;63(8):1637–47. doi: 10.1007/s00125-020-05162-z (PMC7351876; doi:10.1007/s00125-020-05162-z)
Supplement: Supplementary file 1 — (PDF 2.13 mb) [file 125_2020_5162_MOESM1_ESM.pdf]

Electronic supplementary material (ESM)

ESM methods

International Classification of Diseases (ICD) codes used to define the study outcomes

The primary outcomes for this study were major macrovascular and microvascular events and death that occurred during a median of 5 years of follow-up. An independent end point adjudication committee validated all these outcomes. Major macrovascular events were cardiovascular death, nonfatal myocardial infarction or nonfatal stroke

- Death from any cardiovascular cause is a fatal event with an ICD 9 code of 394-459 (diseases of the circulatory system) or 798.9 (sudden death)
- Non-fatal myocardial infarction is a non-fatal event with an ICD 9 code of 410.
- Non-fatal stroke is a non-fatal event with a Ninth International Classification of Diseases (ICD 9) code of 430-435 or 437-438 (i.e. not including transient ischaemic attack). Subarachnoid haemorrhage is not included in the primary outcome definition of stroke).

Major microvascular events were a composite of new or worsening nephropathy or retinopathy, defined as any of the following: (1) development of macroalbuminuria; (2) doubling of serum creatinine level to ≥200 μmol/l; (3) the need for renal replacement therapy due to kidney disease, or death due to renal disease; (4) development of proliferative retinopathy; (5) development of macular oedema; (6) occurrence of diabetes-related blindness; (7) use of retinal photocoagulation therapy.

- New or worsening nephropathy is defined as any of the following:
  - the development of macroalbuminuria (albumin:creatinine ratio >300μg/mg [33.9mg/mmol]), confirmed by two positive results
  - a doubling of serum creatinine to a level of at least 200μmol/l
  - the requirement for renal replacement therapy (dialysis or transplantation)
  - death from renal disease (*All* renal ICD-10 codes are included as potential cause of renal death: N00-N02, NO5 (nephritic syndromes, including haematuria); N04, N06 (nephrotic syndrome, other specified causes of proteinuria); N07 (hereditary nephropathy); N08 (other glomerular disorders); N10, N11, N12 (acute and chronic interstitial nephritis); N13 (obstructive and reflux

uropathy); N14 (drug- and heavy metal induced- interstitial and tubular conditions); N15 (other renal tubulo-interstitial diseases); N17-N19 (acute or chronic renal failure); N20-N23 (urolithiasis); N25 (disorders resulting from impaired renal tubular function); N26 (unspecified contracted kidney); N27 (small kidney of unknown size); N28 (ischaemia of kidney; other unspecified renal disorders); N29 (other disorders of the kidney and ureter in diseases classified elsewhere).

- New or worsening eye disease is defined as any of the following:
  - the requirement for retinal photocoagulation therapy,
  - the development of proliferative retinopathy (new blood vessels on the disc or elsewhere, vitreous haemorrhage, pre-retinal haemorrhage, or fibrous proliferations on the disc or elsewhere), in a participant known not to have this condition at entry
  - the development of macular oedema (retinal thickening within one disc diameter of the macular centre), in a participant known not to have this condition at entry
  - the development of diabetes-related blindness in either eye (corrected visual acuity 6/60 or worse, persisting for three months or more and known not to be due to non-diabetic causes as defined above), in a participant known not to have this condition at entry.

R code used for analysis
(also provided at: https://www.dropbox.com/sh/865cdp9h3zshtn3/AABhw1LJE1kDvSacij0fmofCa?dl=0)

```
###############################1 Data Cleaning################################
########Date 190220

library(foreign)
library(Hmisc)
library(survNRI)
library(survival)
library(cchs)
library(rms)
library(boot)


#################read in ADVANCE data###########################################################
######merge registration and outcomes data (11140 people)
reg_glu=read.dta("##########File Path#########")
outcomes_glu=read.dta("##########File Path#########")
reg_out_glu<-merge(reg_glu,outcomes_glu,by="patient_id")


#################################read in fatty acid data#############################
####fatty acids data - 4637 records and 4009 individuals
fa_data_orig=read.csv("##########File Path#########")

#Identify fatty acid data all NA in finnish data (24 records) - then remove to yield 4613
records and 3990 unique individuals
fa_datav1<- fa_data_orig[rowSums(is.na(fa_data_orig[,4:19]))!=16,]
######sort data in v2 file the in v3 file removes duplicates - thus keeping the first row of the
3990 individuals

fa_datav2<- fa_datav1[order(fa_datav1$reg_num),]
fa_datav3<- fa_datav2[!duplicated(fa_datav2$reg_num),]

######merging fatty acids data (after exluding 24 NA records and deduplicated data ) with
ADVANCE yields  3959 unique id's
reg_out_glu_fadata<-merge(reg_out_glu,fa_datav3,by="reg_num")

####case-cohort
##########################################################################################
######################
#####now identify those in fatty acid  and in case-cohort

cc_orig=read.dta("##########File Path#########")
cc_orig$patient_id<-cc_orig$PATIENT_ID
#########extract relevant columns and rename
cc_extractv1 <- cc_orig[,1:4]
cc_extractv2 <- data.frame(patient_id=cc_orig$PATIENT_ID, cc_extractv1, rs=cc_orig$rand_sample,
crp=cc_orig$crp)
#####merge Fatty acid and ADVANCE data with case-cohort data
reg_out_glu_fadata_cc<-merge(reg_out_glu_fadata,cc_extractv2,by="patient_id")

#############Analysis data
##########################################################################################
###############
# use dat2 as merged fatty acid, case-cohort and ADVANCE data - with 3576 individuals
#####################################################
dat2 <- reg_out_glu_fadata_cc



######3576 case-cohort in total - 2507 controls + 1069 cases (514 from rs and 555 additional)
###rs = 2507 controls and 514 cases


#########additional variables

dat2$TotFA_1sd <- dat2$TotFA/sd(dat2$TotFA)
dat2$UnSat_1sd <- dat2$UnSat/sd(dat2$UnSat)
dat2$DHA_1sd   <- dat2$DHA/sd(dat2$DHA)
dat2$LA_1sd    <- dat2$LA/sd(dat2$LA)
dat2$FAw3_1sd  <- dat2$FAw3/sd(dat2$FAw3)
dat2$FAw6_1sd  <- dat2$FAw6/sd(dat2$FAw6)
dat2$PUFA_1sd  <- dat2$PUFA/sd(dat2$PUFA)
dat2$MUFA_1sd  <- dat2$MUFA/sd(dat2$MUFA)
dat2$SFA_1sd   <- dat2$SFA/sd(na.omit(dat2$SFA))

dat2$DHA.FA_1sd   <- dat2$DHA.FA/sd(dat2$DHA.FA)
```

```
dat2$LA.FA_1sd    <- dat2$LA.FA/sd(dat2$LA.FA)
dat2$FAw3.FA_1sd  <- dat2$FAw3.FA/sd(dat2$FAw3.FA)
dat2$FAw6.FA_1sd  <- dat2$FAw6.FA/sd(dat2$FAw6.FA)
dat2$PUFA.FA_1sd  <- dat2$PUFA.FA/sd(dat2$PUFA.FA)
dat2$MUFA.FA_1sd  <- dat2$MUFA.FA/sd(dat2$MUFA.FA)
dat2$SFA.FA_1sd   <- dat2$SFA.FA/sd(na.omit(dat2$SFA.FA))

######
dat2$ex_mild2[dat2$exercise_mild==0] <- 0
dat2$ex_mild2[dat2$exercise_mild>0] <- 1

dat2$ex_modr2[dat2$exercise_modr==0] <- 0
dat2$ex_modr2[dat2$exercise_modr>0] <- 1

dat2$ex_vig2[dat2$exercise_vig==0] <- 0
dat2$ex_vig2[dat2$exercise_vig>0] <- 1

dat2$ex_comb2[dat2$ex_modr2==0 & dat2$ex_vig2==0] <- 0
dat2$ex_comb2[dat2$ex_modr2==1 & dat2$ex_vig2==1] <- 1
dat2$ex_comb2[dat2$ex_modr2==1 & dat2$ex_vig2==0] <- 1
dat2$ex_comb2[dat2$ex_modr2==0 & dat2$ex_vig2==1] <- 1

dat2$asp[dat2$curr_aspirin==0 | dat2$curr_antiplatelet_other==0] <- 0
dat2$asp[dat2$curr_aspirin==1 | dat2$curr_antiplatelet_other==1] <- 1

dat2$stat[dat2$curr_hmg_coa==0 | dat2$curr_chol_other==0] <- 0
dat2$stat[dat2$curr_hmg_coa==1 | dat2$curr_chol_other==1] <- 1

dat2$ace[dat2$curr_ace_other==0 &  dat2$curr_angio_ii==0 ] <- 0
dat2$ace[dat2$curr_ace_other==1 &  dat2$curr_angio_ii==0 ] <- 1
dat2$ace[dat2$curr_ace_other==0 &  dat2$curr_angio_ii==1 ] <- 1
dat2$ace[dat2$curr_ace_other==1 &  dat2$curr_angio_ii==1 ] <- 1
dat2$ace[dat2$curr_ace_other==0 &  dat2$curr_angio_ii==0 ] <- 0
dat2$ace[dat2$curr_ace_other==1 &  dat2$curr_angio_ii==0 ] <- 1
dat2$ace[dat2$curr_ace_other==0 &  dat2$curr_angio_ii==1 ] <- 1
dat2$ace[dat2$curr_ace_other==1 &  dat2$curr_angio_ii==1 ] <- 1

dat2$aa[dat2$ethnic_code==11] <- 1
dat2$aa[dat2$ethnic_code!=11] <- 0

dat2$trtall=as.factor(dat2$treatment_group_id)

dat2$creatinine_con <-dat2$creatinine*0.0113

#####calculate EGFR in dat2

a=((dat2$creatinine_con)^-1.154)
b=((dat2$age)^-0.203)
c=(0.742*dat2$sex)
c[c==0] <- 1
d=(1.212*dat2$aa)
d[d==0] <- 1
EGFR <-175*a*b*c*d

dat2$egfr <-EGFR

##### subset random subcohort from cohort 3021
randsub<- subset(dat2, dat2$rs==1)


###################2 Hazard ratios proportional hazards regression model to case-cohort
data##########################################################################

#######select one of 3 outcome here- run all models per outcome
####set up for case cohort models - fully adjusted

dat3 <-data.frame(stime= as.numeric(dat2$days_major_macrovascular),
status=as.numeric(dat2$major_macrovascular),y1=as.numeric(dat2$age),y2=dat2$region_name,y3=dat2$
sex,y4=as.factor(dat2$treatment_group_id))

dat3 <-data.frame(stime= as.numeric(dat2$days_microvascular),
status=as.numeric(dat2$microvascular),y1=as.numeric(dat2$age),y2=dat2$region_name,y3=dat2$sex,y4
=as.factor(dat2$treatment_group_id))

dat3<-data.frame(stime= as.numeric(dat2$days_death),
status=as.numeric(dat2$death),y1=as.numeric(dat2$age),y2=dat2$region_name,y3=dat2$sex,y4=as.fact
or(dat2$treatment_group_id))

###############################add additional variables to data frame
dat3$rs <-dat2$rs
dat3$patient_id <-dat2$patient_id
```

```
dat3$TotFA_1sd <- dat2$TotFA_1sd
dat3$UnSat_1sd <- dat2$UnSat_1sd
dat3$DHA_1sd   <- dat2$DHA_1sd
dat3$LA_1sd    <- dat2$LA_1sd
dat3$FAw3_1sd  <- dat2$FAw3_1sd
dat3$FAw6_1sd  <- dat2$FAw6_1sd
dat3$PUFA_1sd  <- dat2$PUFA_1sd
dat3$MUFA_1sd  <- dat2$MUFA_1sd
dat3$SFA_1sd   <- dat2$SFA_1sd

dat3$FAw3.FA  <- dat2$FAw3.FA
dat3$FAw6.FA  <- dat2$FAw6.FA
dat3$PUFA.FA  <- dat2$PUFA.FA
dat3$LA.FA   <- dat2$LA.FA
dat3$DHA.FA  <- dat2$DHA.FA
dat3$MUFA.FA  <- dat2$MUFA.FA
dat3$SFA.FA  <- dat2$SFA.FA

dat3$FAw3.FA_1sd  <- dat2$FAw3.FA_1sd
dat3$FAw6.FA_1sd  <- dat2$FAw6.FA_1sd
dat3$PUFA.FA_1sd  <- dat2$PUFA.FA_1sd
dat3$LA.FA_1sd   <- dat2$LA.FA_1sd
dat3$DHA.FA_1sd  <- dat2$DHA.FA_1sd
dat3$MUFA.FA_1sd  <- dat2$MUFA.FA_1sd
dat3$SFA.FA_1sd  <- dat2$SFA.FA_1sd



dat3$y5  <- dat2$history_macro
dat3$y6  <- dat2$diab_duration
dat3$y7       <- dat2$curr_cig
dat3$y8            <- dat2$sbp
dat3$y9            <- dat2$bmi
dat3$y10       <- dat2$ac_ratio
dat3$y11           <- dat2$egfr
dat3$y12          <- dat2$hba1c
dat3$y15       <- dat2$hdl_chol
dat3$y16       <- dat2$triglyc
dat3$y17            <- dat2$asp
dat3$y18          <- dat2$stat
dat3$y19      <- dat2$curr_beta
dat3$y20           <- dat2$ace


dat3=na.omit(dat3)


#############################################

#######The Prentice method was used for subcohort members at risk and failures (cases) that
occurred outside of the subcohort. ########
#####Dropping those that are censored but not in random subcohort - these need to be dropped
retrospecively otheriwse the models wont run ##################
dat3$drop2[dat3$status==0 & dat3$rs==0] <- 1
dat3$drop2[dat3$status==1 & dat3$rs==0] <- 0
dat3$drop2[dat3$status==0 & dat3$rs==1] <- 0
dat3$drop2[dat3$status==1 & dat3$rs==1] <- 0

dat4 <- subset(dat3, dat3$drop2==0)


###create survival object
surv_object2 <- Surv(time = dat4$stime, event = dat4$status)


########################################fully adjusted##########################################
#######################################Models for % FA per SD increase ####################
p1a =
cch(surv_object2~PUFA.FA_1sd+y1+y2+y3+y4+y5+y6+y7+y8+y9+y10+y11+y12+y15+y16+y17+y18+y19+y20,
data=dat4, id=~patient_id, subcoh=~rs, cohort.size=11140)
summary(p1a)
p1b =
cch(surv_object2~FAw3.FA_1sd+y1+y2+y3+y4+y5+y6+y7+y8+y9+y10+y11+y12+y15+y16+y17+y18+y19+y20,
data=dat4, id=~patient_id, subcoh=~rs, cohort.size=11140)
summary(p1b)
p1c =
cch(surv_object2~DHA.FA_1sd+y1+y2+y3+y4+y5+y6+y7+y8+y9+y10+y11+y12+y15+y16+y17+y18+y19+y20,
data=dat4, id=~patient_id, subcoh=~rs, cohort.size=11140)
summary(p1c)
```

```
p1d =
cch(surv_object2~FAw6.FA_1sd+y1+y2+y3+y4+y5+y6+y7+y8+y9+y10+y11+y12+y15+y16+y17+y18+y19+y20,
data=dat4, id=~patient_id, subcoh=~rs, cohort.size=11140)
summary(p1d)
p1e = cch(surv_object2~LA.FA_1sd+y1+y2+y3+y4+y5+y6+y7+y8+y9+y10+y11+y12+y15+y16+y17+y18+y19+y20,
data=dat4, id=~patient_id, subcoh=~rs, cohort.size=11140)
summary(p1e)
p1f =
cch(surv_object2~MUFA.FA_1sd+y1+y2+y3+y4+y5+y6+y7+y8+y9+y10+y11+y12+y15+y16+y17+y18+y19+y20,
data=dat4, id=~patient_id, subcoh=~rs, cohort.size=11140)
summary(p1f)
p1g =
cch(surv_object2~SFA.FA_1sd+y1+y2+y3+y4+y5+y6+y7+y8+y9+y10+y11+y12+y15+y16+y17+y18+y19+y20,
data=dat4, id=~patient_id, subcoh=~rs, cohort.size=11140)
summary(p1g)



#################basic adjustment

u1a = cch(surv_object2~PUFA.FA_1sd+y1+y2+y3+y4, data=dat4, id=~patient_id, subcoh=~rs,
cohort.size=11140)
summary(u1a)
u1b = cch(surv_object2~FAw3.FA_1sd+y1+y2+y3+y4, data=dat4, id=~patient_id, subcoh=~rs,
cohort.size=11140)
summary(u1b)
u1c = cch(surv_object2~DHA.FA_1sd+y1+y2+y3+y4, data=dat4, id=~patient_id, subcoh=~rs,
cohort.size=11140)
summary(u1c)
u1d = cch(surv_object2~FAw6.FA_1sd+y1+y2+y3+y4, data=dat4, id=~patient_id, subcoh=~rs,
cohort.size=11140)
summary(u1d)
u1e = cch(surv_object2~LA.FA_1sd+y1+y2+y3+y4, data=dat4, id=~patient_id, subcoh=~rs,
cohort.size=11140)
summary(u1e)
u1f = cch(surv_object2~MUFA.FA_1sd+y1+y2+y3+y4, data=dat4, id=~patient_id, subcoh=~rs,
cohort.size=11140)
summary(u1f)
u1g = cch(surv_object2~SFA.FA_1sd+y1+y2+y3+y4, data=dat4, id=~patient_id, subcoh=~rs,
cohort.size=11140)
summary(u1g)

#############3 FULLY ADJUSTED MODEL - cstat##########################################

#######using refined coefficient list : Model 2 included age, sex, region, randomized treatment,
history of macrovascular disease, duration of diabetes, current smoking, systolic blood
pressure, body mass index, urinary albumin-to-creatinine ratio, eGFR, HbA1c, HDL cholesterol,
Triglycerides, aspirin or other antiplatelet agent, statin or other lipid-lowering agent,
betablocker, ACE inhibitor or angiotensin receptor

#####cstatistic calculated on random sub cohort data
#########set up dataframe for analysis

randsub2 <-data.frame(age=randsub$age, sex=randsub$sex, region_name=randsub$region_name,
trtall=as.factor(randsub$treatment_group_id))

###################3 outcomes major macro vascular, major microvascular and death
######################change time and event here for 3 outcomes - run each
separate################################################
#randsub2$time   <- randsub$days_major_macrovascular
#randsub2$event  <- randsub$major_macrovascular

#randsub2$time   <- randsub$days_microvascular
#randsub2$event  <- randsub$microvascular

randsub2$time   <- randsub$days_death
randsub2$event  <- randsub$death
#########################################################################
randsub2$TotFA_1sd <- randsub$TotFA_1sd
randsub2$UnSat_1sd <- randsub$UnSat_1sd
randsub2$DHA_1sd   <- randsub$DHA_1sd
randsub2$LA_1sd    <- randsub$LA_1sd
randsub2$FAw3_1sd  <- randsub$FAw3_1sd
randsub2$FAw6_1sd  <- randsub$FAw6_1sd
randsub2$PUFA_1sd  <- randsub$PUFA_1sd
randsub2$MUFA_1sd  <- randsub$MUFA_1sd
randsub2$SFA_1sd   <- randsub$SFA_1sd

randsub2$FAw3.FA  <- randsub$FAw3.FA
randsub2$FAw6.FA  <- randsub$FAw6.FA
randsub2$PUFA.FA  <- randsub$PUFA.FA
```

```
randsub2$LA.FA   <- randsub$LA.FA
randsub2$DHA.FA  <- randsub$DHA.FA
randsub2$MUFA.FA  <- randsub$MUFA.FA
randsub2$SFA.FA  <- randsub$SFA.FA


randsub2$FAw3.FA_1sd  <- randsub$FAw3.FA_1sd
randsub2$FAw6.FA_1sd  <- randsub$FAw6.FA_1sd
randsub2$PUFA.FA_1sd  <- randsub$PUFA.FA_1sd
randsub2$LA.FA_1sd   <- randsub$LA.FA_1sd
randsub2$DHA.FA_1sd  <- randsub$DHA.FA_1sd
randsub2$MUFA.FA_1sd  <- randsub$MUFA.FA_1sd
randsub2$SFA.FA_1sd  <- randsub$SFA.FA_1sd

randsub2$history_macro  <- randsub$history_macro
randsub2$diab_duration  <- randsub$diab_duration
randsub2$curr_cig       <- randsub$curr_cig
randsub2$sbp            <- randsub$sbp
randsub2$bmi            <- randsub$bmi
randsub2$ac_ratio       <- randsub$ac_ratio
randsub2$egfr           <- randsub$egfr
randsub2$hba1c          <- randsub$hba1c
randsub2$hdl_chol       <- randsub$hdl_chol
randsub2$triglyc        <- randsub$triglyc
randsub2$asp            <- randsub$asp
randsub2$stat           <- randsub$stat
randsub2$curr_beta      <- randsub$curr_beta
randsub2$ace            <- randsub$ace


#age+sex+region_name+trtall+history_macro+diab_duration+curr_cig+sbp+bmi+ac_ratio+egfr+hba1c+hdl
_chol+triglyc+asp+stat+curr_beta+ace
randsub2=na.omit(randsub2)
######################
surv_object3 <- Surv(time = randsub2$time, event = randsub2$event)
surv_object3_rev <- Surv(time = randsub2$time, event = 1-randsub2$event)

############################# fit 6 basic model
fit6.cox <-
coxph(surv_object3~age+sex+region_name+trtall+history_macro+diab_duration+curr_cig+sbp+bmi+ac_ra
tio+egfr+hba1c+hdl_chol+triglyc+asp+stat+curr_beta+ace,data=randsub2)

fit6.coxv2 <- coxph(surv_object3~age+sex+region_name+trtall, data=randsub2, x=TRUE)
fit6cens1 <-
coxph(surv_object3_rev~age+sex+region_name+trtall+history_macro+diab_duration+curr_cig+sbp+bmi+a
c_ratio+egfr+hba1c+hdl_chol+triglyc+asp+stat+curr_beta+ace, data=randsub2)
fit6cens2 <- coxph(surv_object3_rev~1, data=randsub2)
fit6.surv <-survfit(fit6.cox)

formula=surv_object3~age+sex+region_name+trtall+history_macro+diab_duration+curr_cig+sbp+bmi+ac_
ratio+egfr+hba1c+hdl_chol+triglyc+asp+stat+curr_beta+ace

f_fun <- function(d){
  coxph(Surv(time,
event)~age+sex+region_name+trtall+history_macro+diab_duration+curr_cig+sbp+bmi+ac_ratio+egfr+hba
1c+hdl_chol+triglyc+asp+stat+curr_beta+ace, data=d)$concordance[6]}
set.seed(400)
fit6.boot<-censboot(data=randsub2, statistic=f_fun, R=999, F.surv=fit6.surv, G.surv=fit6cens1,
sim="ordinary", cox=fit6.cox, index=c(1,2))
plot(fit6.boot)
fit6.cox$concordance[6]
boot.ci(fit6.boot, type=c("basic", "perc"))

###############################################################
###############################################################
####add additional variables percent fatty acid per 1sd increase
######change variable by model
randsub2$var_fa  <- randsub2$FAw3.FA_1sd
randsub2$var_fa  <- randsub2$DHA.FA_1sd
randsub2$var_fa  <- randsub2$FAw6.FA_1sd
randsub2$var_fa  <- randsub2$PUFA.FA_1sd
randsub2$var_fa  <- randsub2$LA.FA_1sd
randsub2$var_fa  <- randsub2$MUFA.FA_1sd
randsub2$var_fa  <- randsub2$SFA.FA_1sd
randsub2$var_fa  <- randsub2$TotFA_1sd


############perpercent c statistics
set.seed(400)
```

```
fit7.cox <-
coxph(surv_object3~var_fa+age+sex+region_name+trtall+history_macro+diab_duration+curr_cig+sbp+bm
i+ac_ratio+egfr+hba1c+hdl_chol+triglyc+asp+stat+curr_beta+ace, data=randsub2)
fit7.coxv2 <-
coxph(surv_object3~var_fa+age+sex+region_name+trtall+history_macro+diab_duration+curr_cig+sbp+bm
i+ac_ratio+egfr+hba1c+hdl_chol+triglyc+asp+stat+curr_beta+ace, data=randsub2, x=TRUE)

fit7cens1 <-
coxph(surv_object3_rev~var_fa+age+sex+region_name+trtall+history_macro+diab_duration+curr_cig+sb
p+bmi+ac_ratio+egfr+hba1c+hdl_chol+triglyc+asp+stat+curr_beta+ace, data=randsub2)
fit7cens2 <- coxph(surv_object3_rev~1, data=randsub2)
fit7.surv <-survfit(fit7.cox)

formula=surv_object3~var_fa+age+sex+region_name+trtall+history_macro+diab_duration+curr_cig+sbp+
bmi+ac_ratio+egfr+hba1c+hdl_chol+triglyc+asp+stat+curr_beta+ace

f_fun <- function(d){
  coxph(Surv(time,
event)~var_fa+age+sex+region_name+trtall+history_macro+diab_duration+curr_cig+sbp+bmi+ac_ratio+e
gfr+hba1c+hdl_chol+triglyc+asp+stat+curr_beta+ace, data=d)$concordance[6]}

fit7.boot<-censboot(data=randsub2, statistic=f_fun, R=999, F.surv=fit7.surv, G.surv=fit7cens1,
sim="ordinary", cox=fit7.cox, index=c(1,2))
plot(fit7.boot)
fit7.cox$concordance[6]
boot.ci(fit7.boot, type=c("basic", "perc"))

##difference in c-statistics compare concordance values
ctest <- concordance(fit6.cox, fit7.cox)
contr <- c(-1,1)
dtest <- contr %*% coef(ctest)
dvar <- contr %*% vcov(ctest) %*% contr
#c(contrast=dtest, sd=sqrt(dvar), z=dtest/sqrt(dvar))

con1=c(contrast=dtest, sd=sqrt(dvar), z=dtest/sqrt(dvar))["contrast"]
sdcon=c(contrast=dtest, sd=sqrt(dvar), z=dtest/sqrt(dvar))["sd"]
cbind(con=con1,lcon=con1-1.96*(sdcon), ucon=con1+1.96*(sdcon) )
cbind(con=round(con1,4),lcon=round(con1-1.96*(sdcon),4), ucon=round(con1+1.96*(sdcon),4) )

###############################################################################################
################################################################
##############################basic model adjustment##################################

randsub2 <-data.frame(age=randsub$age, sex=randsub$sex, region_name=randsub$region_name,
trtall=as.factor(randsub$treatment_group_id))

###################3 outcomes major macro vascular, major microvascular and death
######################change time and event here for 3 outcomes - run each
separate##################################################
#randsub2$time   <- randsub$days_major_macrovascular
#randsub2$event  <- randsub$major_macrovascular

#randsub2$time   <- randsub$days_microvascular
#randsub2$event  <- randsub$microvascular

randsub2$time   <- randsub$days_death
randsub2$event  <- randsub$death
###########################################################################
randsub2$TotFA_1sd <- randsub$TotFA_1sd
randsub2$UnSat_1sd <- randsub$UnSat_1sd
randsub2$DHA_1sd   <- randsub$DHA_1sd
randsub2$LA_1sd    <- randsub$LA_1sd
randsub2$FAw3_1sd  <- randsub$FAw3_1sd
randsub2$FAw6_1sd  <- randsub$FAw6_1sd
randsub2$PUFA_1sd  <- randsub$PUFA_1sd
randsub2$MUFA_1sd  <- randsub$MUFA_1sd
randsub2$SFA_1sd   <- randsub$SFA_1sd

randsub2$FAw3.FA  <- randsub$FAw3.FA
randsub2$FAw6.FA  <- randsub$FAw6.FA
randsub2$PUFA.FA  <- randsub$PUFA.FA
randsub2$LA.FA   <- randsub$LA.FA
randsub2$DHA.FA  <- randsub$DHA.FA
randsub2$MUFA.FA  <- randsub$MUFA.FA
randsub2$SFA.FA  <- randsub$SFA.FA

randsub2$FAw3.FA_1sd  <- randsub$FAw3.FA_1sd
randsub2$FAw6.FA_1sd  <- randsub$FAw6.FA_1sd
randsub2$PUFA.FA_1sd  <- randsub$PUFA.FA_1sd
randsub2$LA.FA_1sd   <- randsub$LA.FA_1sd
randsub2$DHA.FA_1sd  <- randsub$DHA.FA_1sd
```

```
randsub2$MUFA.FA_1sd  <- randsub$MUFA.FA_1sd
randsub2$SFA.FA_1sd  <- randsub$SFA.FA_1sd

randsub2=na.omit(randsub2)
######################
surv_object3 <- Surv(time = randsub2$time, event = randsub2$event)
surv_object3_rev <- Surv(time = randsub2$time, event = 1-randsub2$event)

############################## fit 6 basic model #age+sex+region_name+trtall
fit6.cox <- coxph(surv_object3~age+sex+region_name+trtall,data=randsub2)

fit6.coxv2 <- coxph(surv_object3~age+sex+region_name+trtall, data=randsub2, x=TRUE)
fit6cens1 <- coxph(surv_object3_rev~age+sex+region_name+trtall, data=randsub2)
fit6cens2 <- coxph(surv_object3_rev~1, data=randsub2)
fit6.surv <-survfit(fit6.cox)

formula=surv_object3~age+sex+region_name+trtall

f_fun <- function(d){
  coxph(Surv(time, event)~age+sex+region_name+trtall, data=d)$concordance[6]}
set.seed(400)
fit6.boot<-censboot(data=randsub2, statistic=f_fun, R=999, F.surv=fit6.surv, G.surv=fit6cens1,
sim="ordinary", cox=fit6.cox, index=c(1,2))
plot(fit6.boot)
fit6.cox$concordance[6]
boot.ci(fit6.boot, type=c("basic", "perc"))

################################################################
################################################################
####add additional variables percent fatty acid per 1sd increase
######change variable by model
randsub2$var_fa  <- randsub2$FAw3.FA_1sd
randsub2$var_fa  <- randsub2$DHA.FA_1sd
randsub2$var_fa  <- randsub2$FAw6.FA_1sd
randsub2$var_fa  <- randsub2$PUFA.FA_1sd
randsub2$var_fa  <- randsub2$LA.FA_1sd
randsub2$var_fa  <- randsub2$MUFA.FA_1sd
randsub2$var_fa  <- randsub2$SFA.FA_1sd
randsub2$var_fa  <- randsub2$TotFA_1sd


#############perpercent c statistics
set.seed(400)


fit7.cox <- coxph(surv_object3~var_fa+age+sex+region_name+trtall, data=randsub2)
fit7.coxv2 <- coxph(surv_object3~var_fa+age+sex+region_name+trtall, data=randsub2, x=TRUE)

fit7cens1 <- coxph(surv_object3_rev~var_fa+age+sex+region_name+trtall, data=randsub2)
fit7cens2 <- coxph(surv_object3_rev~1, data=randsub2)
fit7.surv <-survfit(fit7.cox)

formula=surv_object3~var_fa+age+sex+region_name+trtall

f_fun <- function(d){
  coxph(Surv(time, event)~var_fa+age+sex+region_name+trtall, data=d)$concordance[6]}

fit7.boot<-censboot(data=randsub2, statistic=f_fun, R=999, F.surv=fit7.surv, G.surv=fit7cens1,
sim="ordinary", cox=fit7.cox, index=c(1,2))
plot(fit7.boot)
fit7.cox$concordance[6]
boot.ci(fit7.boot, type=c("basic", "perc"))

##difference in c-statistics compare concordance values
ctest <- concordance(fit6.cox, fit7.cox)
contr <- c(-1,1)
dtest <- contr %*% coef(ctest)
dvar <- contr %*% vcov(ctest) %*% contr
#c(contrast=dtest, sd=sqrt(dvar), z=dtest/sqrt(dvar))

con1=c(contrast=dtest, sd=sqrt(dvar), z=dtest/sqrt(dvar))["contrast"]
sdcon=c(contrast=dtest, sd=sqrt(dvar), z=dtest/sqrt(dvar))["sd"]
cbind(con=con1,lcon=con1-1.96*(sdcon), ucon=con1+1.96*(sdcon) )
cbind(con=round(con1,4),lcon=round(con1-1.96*(sdcon),4), ucon=round(con1+1.96*(sdcon),4) )



#####4 NRI ######################################################################

#library(devtools)
#install_github("mdbrown/survNRI")
```

```
#http://mdbrown.github.io/survNRI/
library(survNRI)

#running for significant fatty acids from CCh models - only omega 3 and dha

#######select outcome here#######


#######major macro
randsub3 <-data.frame(stime= as.numeric(randsub$days_major_macrovascular),
status=as.numeric(randsub$major_macrovascular),y1=as.numeric(randsub$age),y2=randsub$region_name
,y3=randsub$sex,y4=as.factor(randsub$treatment_group_id))


##########add variables to randomsubset

randsub3$TotFA_1sd <- randsub$TotFA_1sd
randsub3$UnSat_1sd <- randsub$UnSat_1sd
randsub3$DHA_1sd   <- randsub$DHA_1sd
randsub3$LA_1sd    <- randsub$LA_1sd
randsub3$FAw3_1sd  <- randsub$FAw3_1sd
randsub3$FAw6_1sd  <- randsub$FAw6_1sd
randsub3$PUFA_1sd  <- randsub$PUFA_1sd
randsub3$MUFA_1sd  <- randsub$MUFA_1sd
randsub3$SFA_1sd   <- randsub$SFA_1sd

randsub3$FAw3.FA  <- randsub$FAw3.FA
randsub3$FAw6.FA  <- randsub$FAw6.FA
randsub3$PUFA.FA  <- randsub$PUFA.FA
randsub3$LA.FA   <- randsub$LA.FA
randsub3$DHA.FA  <- randsub$DHA.FA
randsub3$MUFA.FA  <- randsub$MUFA.FA
randsub3$SFA.FA  <- randsub$SFA.FA

randsub3$FAw3.FA_1sd  <- randsub$FAw3.FA_1sd
randsub3$FAw6.FA_1sd  <- randsub$FAw6.FA_1sd
randsub3$PUFA.FA_1sd  <- randsub$PUFA.FA_1sd
randsub3$LA.FA_1sd   <- randsub$LA.FA_1sd
randsub3$DHA.FA_1sd  <- randsub$DHA.FA_1sd
randsub3$MUFA.FA_1sd  <- randsub$MUFA.FA_1sd
randsub3$SFA.FA_1sd  <- randsub$SFA.FA_1sd


randsub3$y5  <- randsub$history_macro
randsub3$y6  <- randsub$diab_duration
randsub3$y7       <- randsub$curr_cig
randsub3$y8            <- randsub$sbp
randsub3$y9            <- randsub$bmi
randsub3$y10       <- randsub$ac_ratio
randsub3$y11           <- randsub$egfr
randsub3$y12          <- randsub$hba1c
randsub3$y15       <- randsub$hdl_chol
randsub3$y16       <- randsub$triglyc
randsub3$y17            <- randsub$asp
randsub3$y18          <- randsub$stat
randsub3$y19      <- randsub$curr_beta
randsub3$y20           <- randsub$ace

randsub3=na.omit(randsub3)

####################NRI - major macro

Sys.time()

set.seed(400)
survNRI( time  = "stime", event = "status",
         model1 = c("y1","y2","y3","y4", "y5", "y6","y7","y8","y9", "y10","y11","y12", "y15",
"y16","y17","y18","y19", "y20"),
         model2 = c("y1","y2","y3","y4", "y5", "y6","y7","y8","y9", "y10","y11","y12", "y15",
"y16","y17","y18","y19", "y20", "FAw3.FA_1sd"),
         data = randsub3,
         predict.time = 1825,
         method = "all",
         bootMethod = "normal",
         bootstraps = 500)

set.seed(400)
survNRI( time  = "stime", event = "status",
         model1 = c("y1","y2","y3","y4", "y5", "y6","y7","y8","y9", "y10","y11","y12", "y15",
"y16","y17","y18","y19", "y20"),
```

```
         model2 = c("y1","y2","y3","y4", "y5", "y6","y7","y8","y9", "y10","y11","y12", "y15",
"y16","y17","y18","y19", "y20", "DHA.FA_1sd"),
         data = randsub3,
         predict.time = 1825,
         method = "all",
         bootMethod = "normal",
         bootstraps = 500)


Sys.time()

############################################################
#####death models

randsub3 <-data.frame(stime= as.numeric(randsub$days_death),
status=as.numeric(randsub$death),y1=as.numeric(randsub$age),y2=randsub$region_name,y3=randsub$se
x,y4=as.factor(randsub$treatment_group_id))

randsub3$TotFA_1sd <- randsub$TotFA_1sd
randsub3$UnSat_1sd <- randsub$UnSat_1sd
randsub3$DHA_1sd   <- randsub$DHA_1sd
randsub3$LA_1sd    <- randsub$LA_1sd
randsub3$FAw3_1sd  <- randsub$FAw3_1sd
randsub3$FAw6_1sd  <- randsub$FAw6_1sd
randsub3$PUFA_1sd  <- randsub$PUFA_1sd
randsub3$MUFA_1sd  <- randsub$MUFA_1sd
randsub3$SFA_1sd   <- randsub$SFA_1sd

randsub3$FAw3.FA  <- randsub$FAw3.FA
randsub3$FAw6.FA  <- randsub$FAw6.FA
randsub3$PUFA.FA  <- randsub$PUFA.FA
randsub3$LA.FA   <- randsub$LA.FA
randsub3$DHA.FA  <- randsub$DHA.FA
randsub3$MUFA.FA  <- randsub$MUFA.FA
randsub3$SFA.FA  <- randsub$SFA.FA

randsub3$FAw3.FA_1sd  <- randsub$FAw3.FA_1sd
randsub3$FAw6.FA_1sd  <- randsub$FAw6.FA_1sd
randsub3$PUFA.FA_1sd  <- randsub$PUFA.FA_1sd
randsub3$LA.FA_1sd   <- randsub$LA.FA_1sd
randsub3$DHA.FA_1sd  <- randsub$DHA.FA_1sd
randsub3$MUFA.FA_1sd  <- randsub$MUFA.FA_1sd
randsub3$SFA.FA_1sd  <- randsub$SFA.FA_1sd

randsub3$y5  <- randsub$history_macro
randsub3$y6  <- randsub$diab_duration
randsub3$y7       <- randsub$curr_cig
randsub3$y8            <- randsub$sbp
randsub3$y9            <- randsub$bmi
randsub3$y10       <- randsub$ac_ratio
randsub3$y11           <- randsub$egfr
randsub3$y12          <- randsub$hba1c
randsub3$y15       <- randsub$hdl_chol
randsub3$y16       <- randsub$triglyc
randsub3$y17             <- randsub$asp
randsub3$y18          <- randsub$stat
randsub3$y19      <- randsub$curr_beta
randsub3$y20           <- randsub$ace


randsub3=na.omit(randsub3)

#######NRI
Sys.time()


set.seed(400)
survNRI( time  = "stime", event = "status",
         model1 = c("y1","y2","y3","y4", "y5", "y6","y7","y8","y9", "y10","y11","y12", "y15",
"y16","y17","y18","y19", "y20"),
         model2 = c("y1","y2","y3","y4", "y5", "y6","y7","y8","y9", "y10","y11","y12", "y15",
"y16","y17","y18","y19", "y20", "FAw3.FA_1sd"),
         data = randsub3,
         predict.time = 1825,
         method = "all",
         bootMethod = "normal",
         bootstraps = 500)

set.seed(400)
survNRI( time  = "stime", event = "status",
```

```
        model1 = c("y1","y2","y3","y4", "y5", "y6","y7","y8","y9", "y10","y11","y12", "y15", 
"y16","y17","y18","y19", "y20"),
        model2 = c("y1","y2","y3","y4", "y5", "y6","y7","y8","y9", "y10","y11","y12", "y15", 
"y16","y17","y18","y19", "y20", "DHA.FA_1sd"),
        data = randsub3,
        predict.time = 1825,
        method = "all",
        bootMethod = "normal",
        bootstraps = 500)

Sys.time()
##################################
```

ESM tables

ESM table 1: Adjusted hazard ratios of omega-3 fatty acids and docosahexaenoic acid (DHA) (per 1SD increase of percentage of total fatty acids) for macrovascular events, death and microvascular events according to baseline subgroups of history of microvascular disease from multiple adjusted models*

**History of microvascular disease**	**Outcome**	**Total**	**HR (95% CI)** n-3 fatty acids	**P for interaction**	**HR (95% CI)** DHA	**P for interaction**
	Major macrovascular					
No	537	3323	0.85 (0.77, 0.95)	*0.297*	0.87 (0.78, 0.96)	*0.613*
Yes	117	353	0.99 (0.75, 1.30)		0.93 (0.71, 1.22)	
	Death					
No	524	3323	0.91 (0.82, 1.01)	*0.761*	0.93 (0.84, 1.04)	*0.538*
Yes	107	353	0.87 (0.65, 1.16)		0.85 (0.64, 1.13)	
	Major microvascular					
No	264	3323	0.94 (0.82, 1.08)	*0.243*	0.94 (0.82, 1.08)	*0.247*
Yes	77	353	1.15 (0.83, 1.59)		1.14 (0.83, 1.57)	

*Adjusted for age, sex, region, randomised treatment, history of macrovascular disease, duration of diabetes, current smoking status, systolic BP, BMI, urinary albumin/creatinine ratio, eGFR, HbA_{1c}, HDL-cholesterol, triacylglycerols, and use of aspirin or other antiplatelet agents, statins or other lipid-lowering agents, β-blockers, and ACE inhibitors or angiotensin receptor blockers

ESM table 2: Prognostic value of fatty acids using C-statistic (and difference) and continuous net reclassification improvement (NRI) with 95% Confidence Intervals (CI) for macrovascular, microvascular and death (per 1SD increase of percentage of total fatty acids) from models adjusted for age, sex, region and randomised treatment (basic model 1).

**Fatty acids (per percentage of total fatty acids)**	**C-statistic and difference**	**95% CI**	**Continuous NRI**	**95% CI**
**Macrovascular events**				
Basic model 1	*0.6291*	*0.5925, 0.6535*	-	-
+ PUFA	+ 0.0035	-0.0037, 0.0107	0.103	0.053, 0.202
+ n-3 fatty acids	+ 0.0107	-0.0008, 0.0223	0.157	-0.006, 0.267
+ DHA	+ 0.0104	0.0001, 0.0206	0.156	-0.009, 0.265
+ LA	+ 0.0015	-0.0043, 0.0072	0.069	-0.063, 0.167
**Microvascular events**				
Basic model 1	*0.6054*	*0.5595, 0.6316*	-	-
+ MUFA	-0.0017	-0.0094, 0.0061	0.011	0.029, 0.129
**Death**				
Basic model 1	*0.6701*	*0.6387, 0.6984*	-	-
+ PUFA	+ 0.0050	-0.0017, 0.0117	0.132	0.044, 0.248
+ n-3 fatty acids	+ 0.0103	0.0004, 0.0202	0.170	-0.013, 0.293
+ DHA	+ 0.0084	0.0000, 0.0169	0.161	-0.008, 0.272
+ LA	+ 0.0044	-0.0020, 0.0108	0.111	0.035, 0.229

ESM figures

ESM fig 1: Adjusted hazard ratios of the fatty acids (per 1SD increase of percentage of total fatty acids) for macrovascular events, microvascular events and death from models adjusted for age, sex, region and randomised treatment

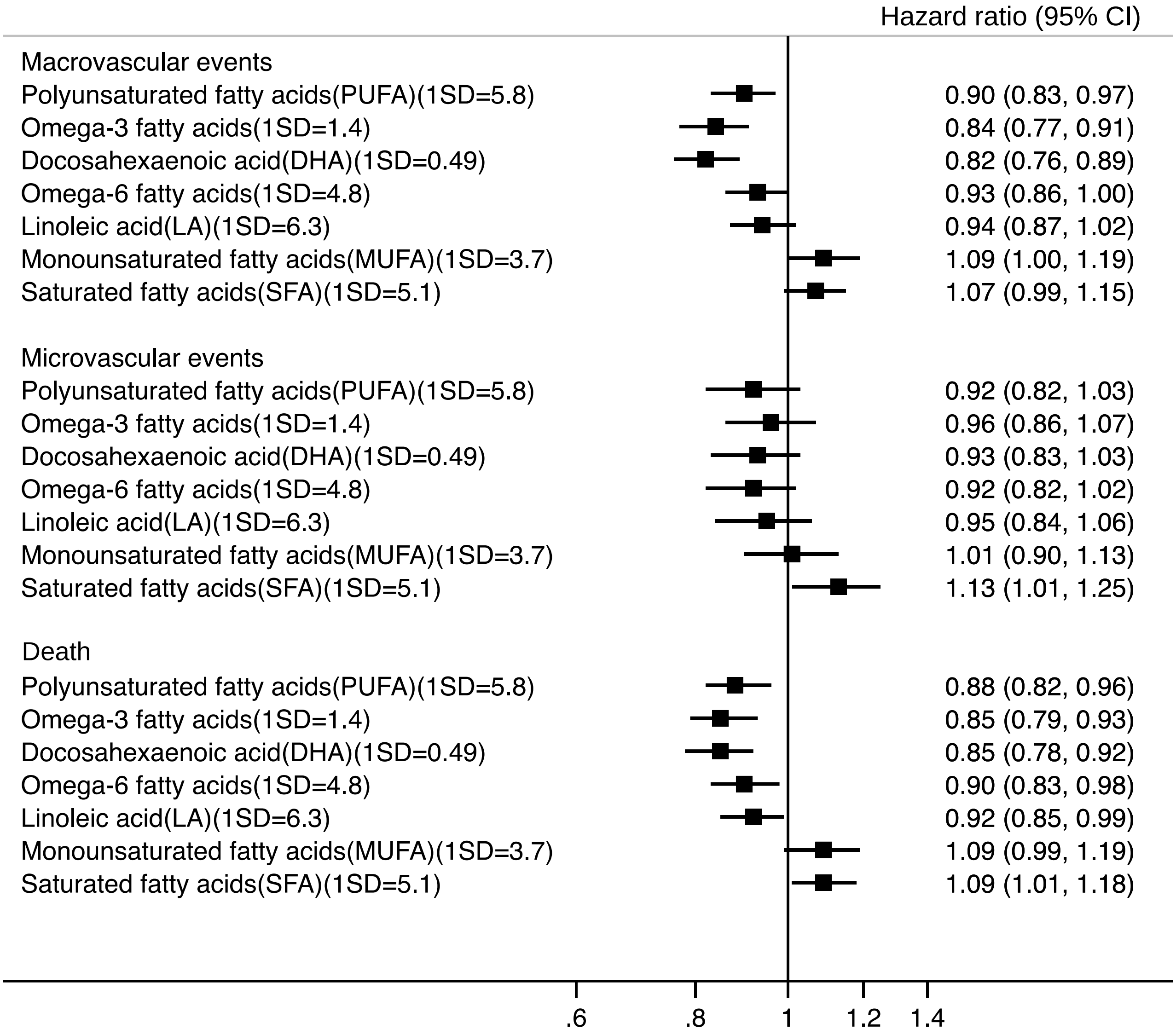

ESM fig 2. Adjusted hazard ratios of the fatty acids (per 1SD increase of percentage of total fatty acids) for new or worsening nephropathy from multiple adjusted models*

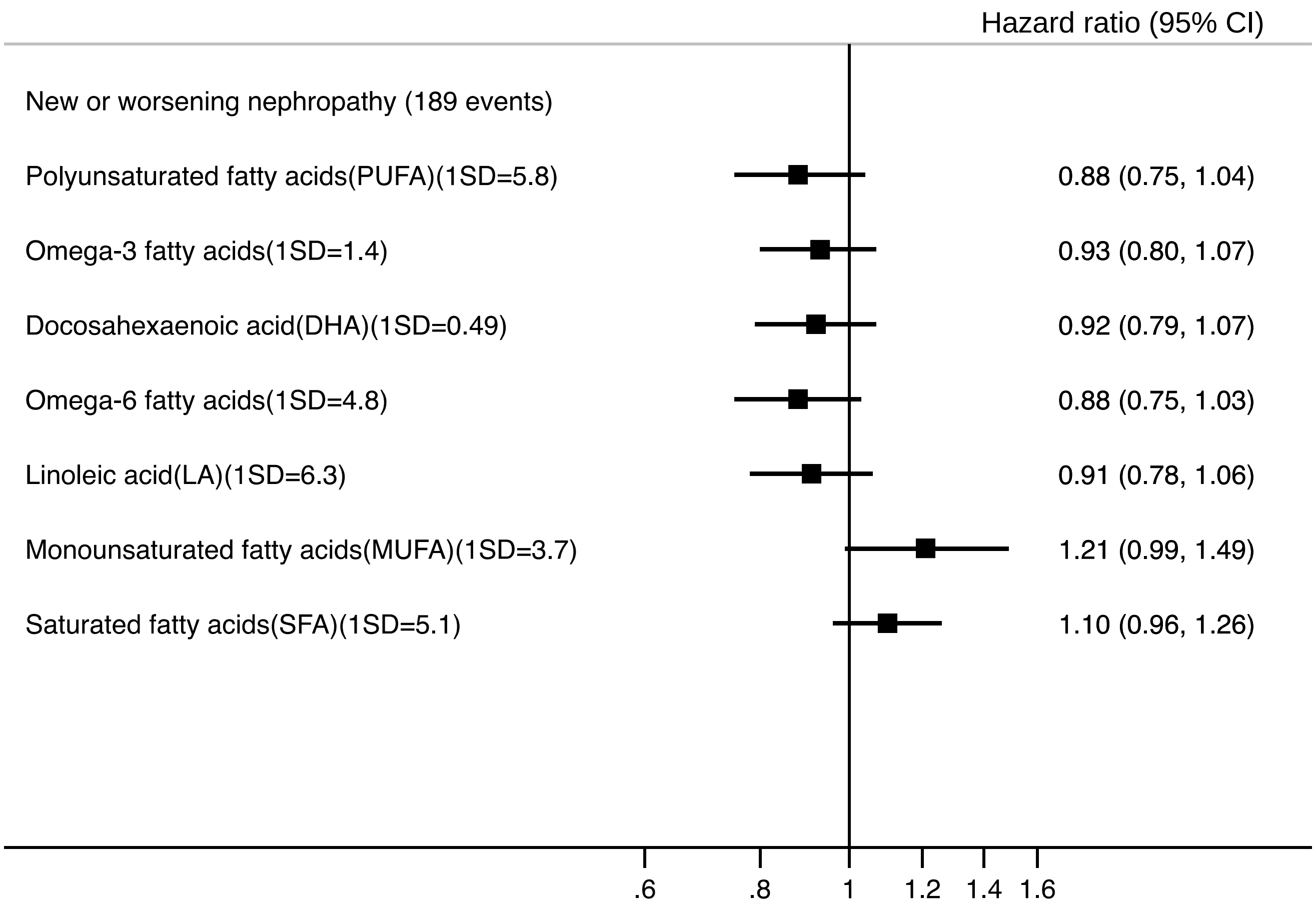

*Adjusted for age, sex, region, randomised treatment, history of macrovascular disease, duration of diabetes, current smoking status, systolic BP, BMI, urinary albumin/creatinine ratio, eGFR, HbA_{1c}, HDL-cholesterol, triacylglycerols, and use of aspirin or other antiplatelet agents, statins or other lipid-lowering agents, β-blockers, and ACE inhibitors or angiotensin receptor blockers

ESM fig 3: Adjusted hazard ratios of omega-3 fatty acids and docosahexaenoic acid (DHA) according to categories for (A) macrovascular events and (B) death from multiple adjusted models*

A

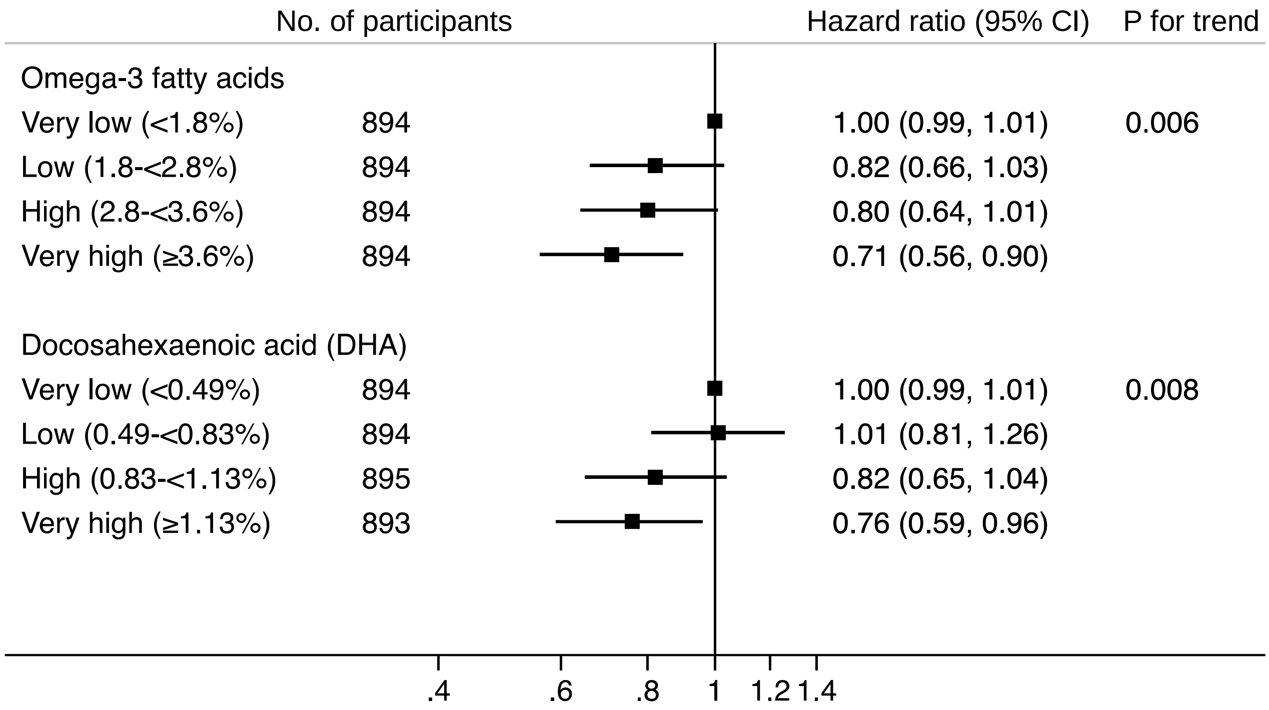

B

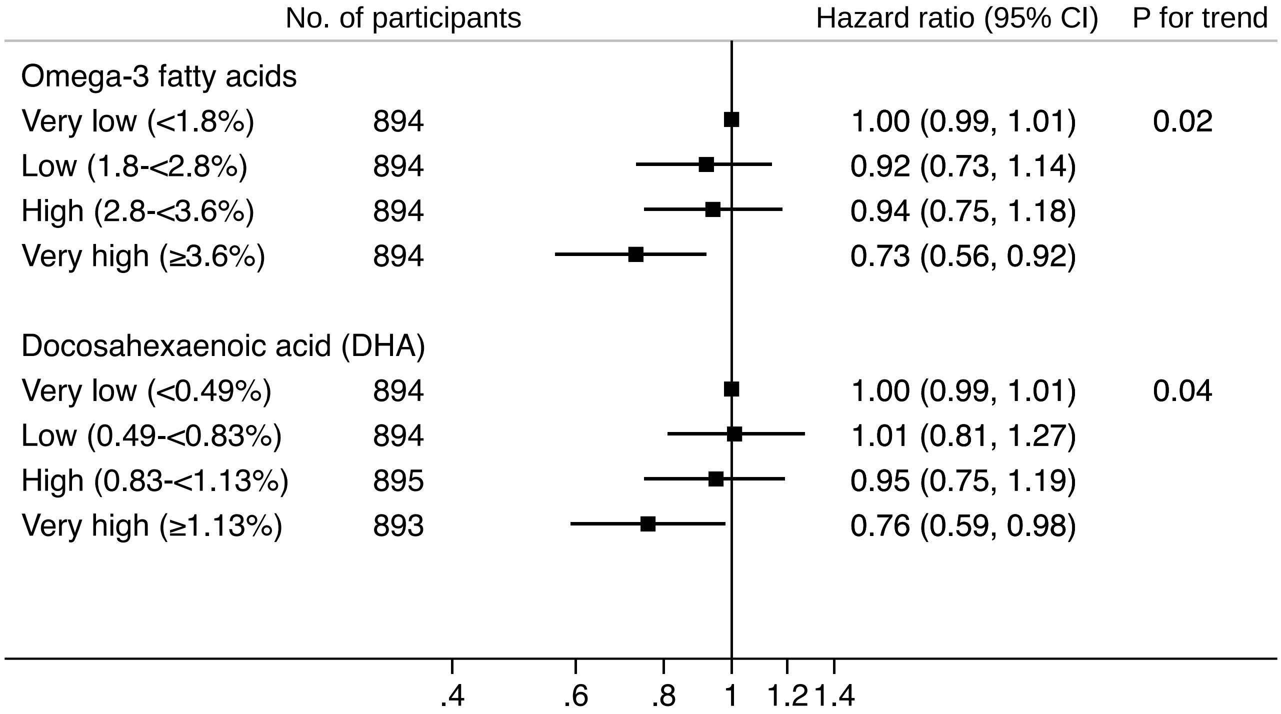

*Adjusted for age, sex, region, randomised treatment, history of macrovascular disease, duration of diabetes, current smoking status, systolic BP, BMI, urinary albumin/creatinine ratio, eGFR, HbA_{1c}, HDL-cholesterol, triacylglycerols, and use of aspirin or other antiplatelet agents, statins or other lipid-lowering agents, β-blockers, and ACE inhibitors or angiotensin receptor blockers

ESM fig 4. Adjusted hazard ratios of the fatty acids for macrovascular events, microvascular events and death (per 1 SD increase of fatty acids) from (A) model 1 and (B) model 2

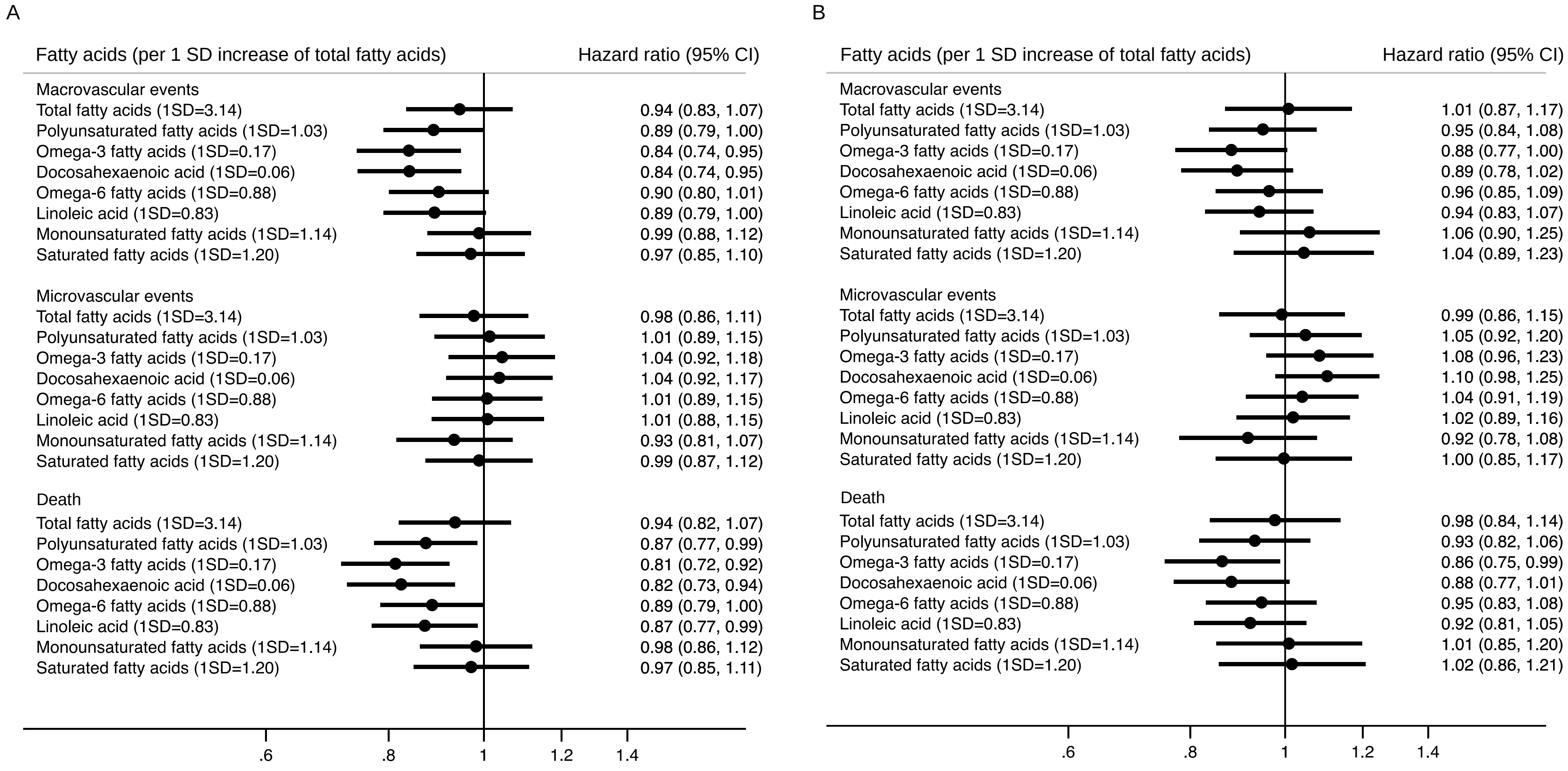

Model 1 Adjusted for age, sex, region and randomised treatment
Model 2 Adjusted for age, sex, region, randomised treatment, history of macrovascular disease, duration of diabetes, current smoking status, systolic BP, BMI, urinary albumin/creatinine ratio, eGFR, HbA_{1c}, HDL-cholesterol, triacylglycerols, and use of aspirin or other antiplatelet agents, statins or other lipid-lowering agents, β-blockers, and ACE inhibitors or angiotensin receptor blockers

ESM fig 5. Adjusted hazard ratios of (A) omega-3 fatty acids and (B) docosahexaenoic acid (DHA) (per 1SD increase of percentage of total fatty acids) for macrovascular events according to baseline subgroups from multiple adjusted models*

A

Subgroup	Number events	Number total	Hazard ratio (95% CI)	P for interaction
Age				
≥65	502	2342	0.90 (0.82, 0.99)	0.24
<65	152	1234	0.75 (0.62, 0.90)	
Sex				
Men	450	2162	0.85 (0.77, 0.94)	0.64
Women	204	1414	0.93 (0.80, 1.08)	
Region				
ANZ/SEA	155	868	0.86 (0.72, 1.01)	1.00
Canada	33	218	0.69 (0.36, 1.32)	
Continental Europe	262	1416	0.88 (0.76, 1.01)	
Northern Europe	204	1074	0.87 (0.74, 1.01)	
History of macrovascular disease				
No	331	2328	0.92 (0.82, 1.04)	0.17
Yes	323	1248	0.83 (0.73, 0.94)	
eGFR (ml/min/1.73 m^2)				
≥60	434	2693	0.87 (0.78, 0.96)	0.99
<60	216	866	0.88 (0.76, 1.03)	
Triglycerides (mmol/l)				
<1.7	337	1769	0.78 (0.69, 0.88)	0.02
≥1.7	317	1802	0.98 (0.87, 1.10)	
Randomised BP lowering treatment				
BP treatment	310	1763	0.96 (0.86, 1.08)	0.01
Placebo	344	1813	0.78 (0.69, 0.88)	
Randomised intensive blood glucose control				
Intensive	321	1766	0.92 (0.82, 1.04)	0.32
Standard	333	1477	0.82 (0.73, 0.93)	

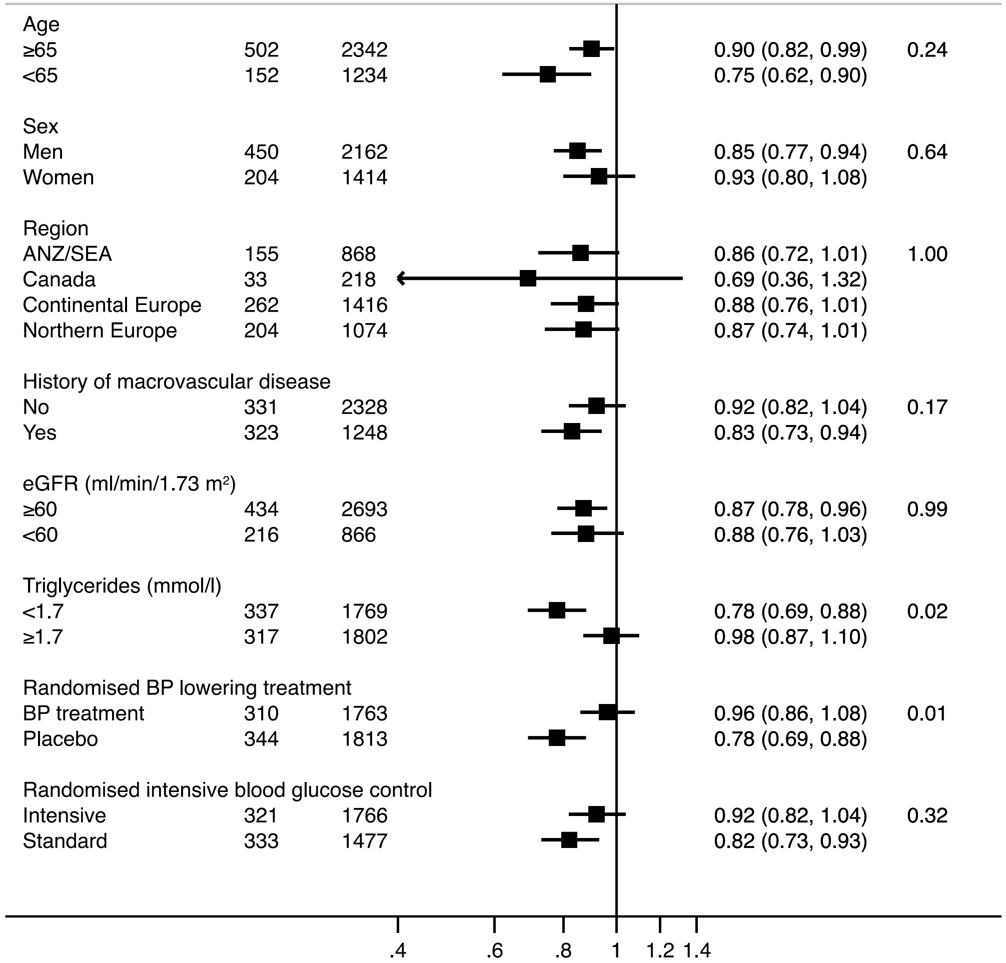

B

Subgroup	Number events	Number total	Hazard ratio (95% CI)	P for interaction
Age				
≥65	502	2342	0.91 (0.83, 1.01)	0.17
<65	152	1234	0.73 (0.61, 0.88)	
Sex				
Men	450	2162	0.86 (0.77, 0.95)	0.54
Women	204	1414	0.95 (0.81, 1.11)	
Region				
ANZ/SEA	155	868	0.86 (0.72, 1.01)	0.75
Canada	33	218	0.74 (0.39, 1.30)	
Continental Europe	262	1416	0.86 (0.74, 0.99)	
Northern Europe	204	1074	0.91 (0.79, 1.06)	
History of macrovascular disease				
No	331	2328	0.91 (0.81, 1.03)	0.48
Yes	323	1248	0.87 (0.76, 0.98)	
eGFR (ml/min/1.73 m^2)				
≥60	434	2693	0.87 (0.78, 0.97)	0.70
<60	216	866	0.90 (0.78, 1.05)	
Triglycerides (mmol/l)				
<1.7	337	1769	0.80 (0.70, 0.90)	0.05
≥1.7	317	1802	0.98 (0.87, 1.11)	
Randomised BP lowering treatment				
BP treatment	310	1763	0.92 (0.82, 1.04)	0.25
Placebo	344	1813	0.84 (0.75, 0.95)	
Randomised intensive blood glucose control				
Intensive	321	1766	0.89 (0.78, 1.01)	0.96
Standard	333	1477	0.87 (0.77, 0.98)	

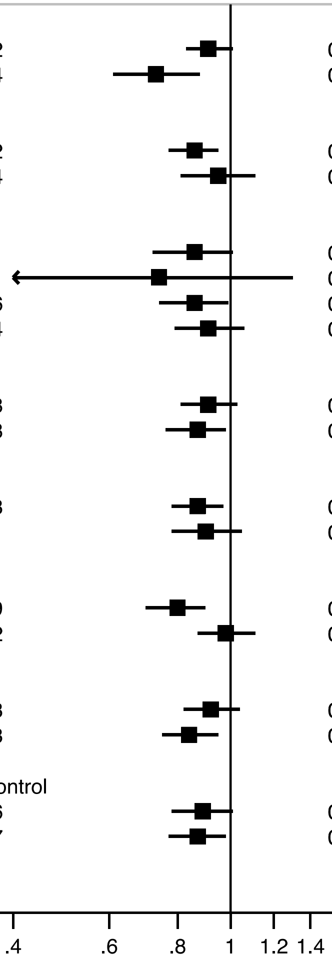

*Adjusted for age, sex, region, randomised treatment, history of macrovascular disease, duration of diabetes, current smoking status, systolic BP, BMI, urinary albumin/creatinine ratio, eGFR, HbA_{1c}, HDL-cholesterol, triacylglycerols, and use of aspirin or other antiplatelet agents, statins or other lipid-lowering agents, β-blockers, and ACE inhibitors or angiotensin receptor blockers

ESM fig 6 Adjusted hazard ratios of (A) omega-3 fatty acids and (B) docosahexaenoic acid (DHA) (per 1SD increase of percentage of total fatty acids) for death according to baseline subgroups from fully adjusted models*.

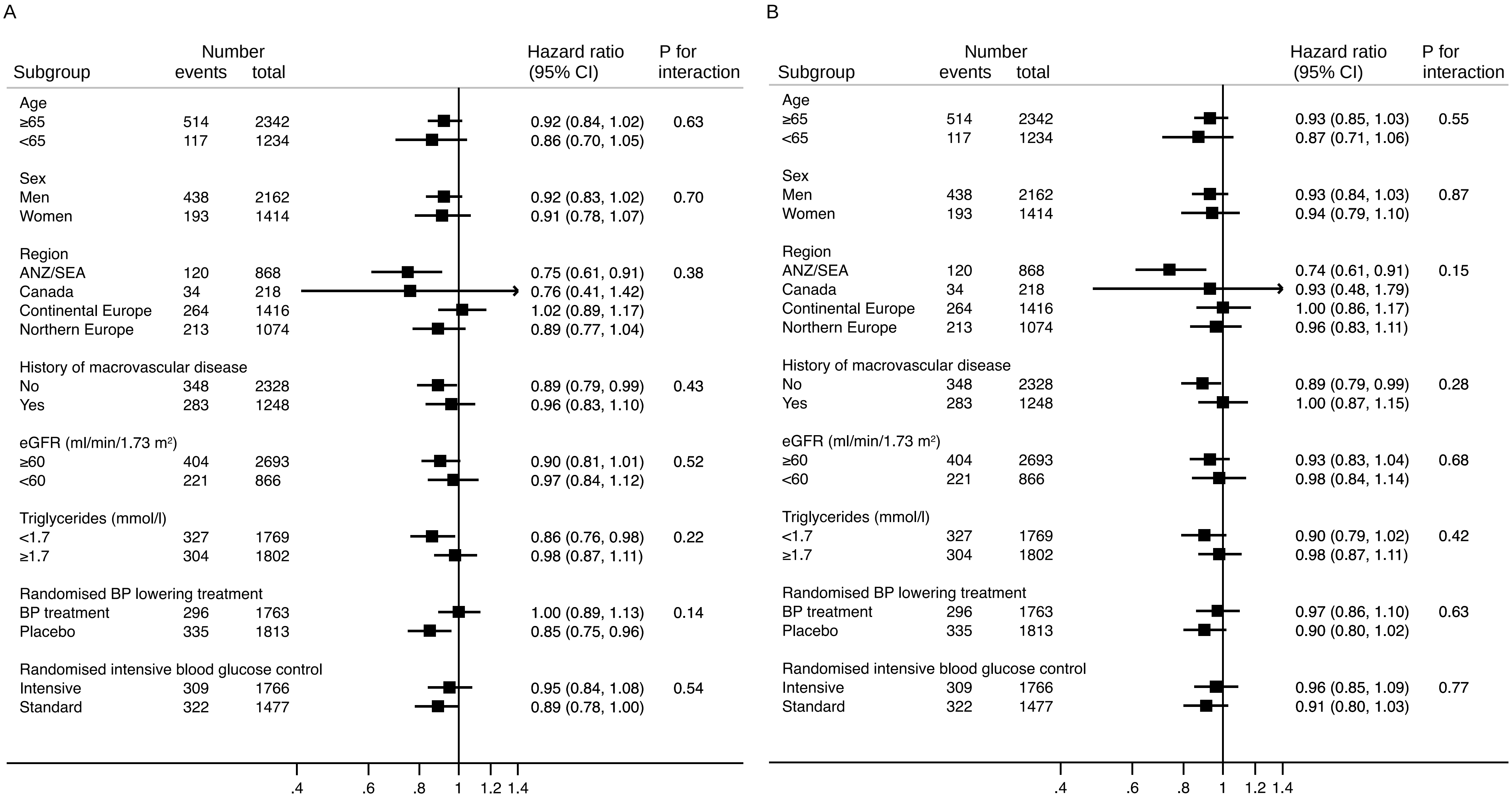

A

Subgroup	Number events	Number total	Hazard ratio (95% CI)	P for interaction
Age				
≥65	514	2342	0.92 (0.84, 1.02)	0.63
<65	117	1234	0.86 (0.70, 1.05)	
Sex				
Men	438	2162	0.92 (0.83, 1.02)	0.70
Women	193	1414	0.91 (0.78, 1.07)	
Region				
ANZ/SEA	120	868	0.75 (0.61, 0.91)	0.38
Canada	34	218	0.76 (0.41, 1.42)	
Continental Europe	264	1416	1.02 (0.89, 1.17)	
Northern Europe	213	1074	0.89 (0.77, 1.04)	
History of macrovascular disease				
No	348	2328	0.89 (0.79, 0.99)	0.43
Yes	283	1248	0.96 (0.83, 1.10)	
eGFR (ml/min/1.73 m^2)				
≥60	404	2693	0.90 (0.81, 1.01)	0.52
<60	221	866	0.97 (0.84, 1.12)	
Triglycerides (mmol/l)				
<1.7	327	1769	0.86 (0.76, 0.98)	0.22
≥1.7	304	1802	0.98 (0.87, 1.11)	
Randomised BP lowering treatment				
BP treatment	296	1763	1.00 (0.89, 1.13)	0.14
Placebo	335	1813	0.85 (0.75, 0.96)	
Randomised intensive blood glucose control				
Intensive	309	1766	0.95 (0.84, 1.08)	0.54
Standard	322	1477	0.89 (0.78, 1.00)	

B

Subgroup	Number events	Number total	Hazard ratio (95% CI)	P for interaction
Age				
≥65	514	2342	0.93 (0.85, 1.03)	0.55
<65	117	1234	0.87 (0.71, 1.06)	
Sex				
Men	438	2162	0.93 (0.84, 1.03)	0.87
Women	193	1414	0.94 (0.79, 1.10)	
Region				
ANZ/SEA	120	868	0.74 (0.61, 0.91)	0.15
Canada	34	218	0.93 (0.48, 1.79)	
Continental Europe	264	1416	1.00 (0.86, 1.17)	
Northern Europe	213	1074	0.96 (0.83, 1.11)	
History of macrovascular disease				
No	348	2328	0.89 (0.79, 0.99)	0.28
Yes	283	1248	1.00 (0.87, 1.15)	
eGFR (ml/min/1.73 m^2)				
≥60	404	2693	0.93 (0.83, 1.04)	0.68
<60	221	866	0.98 (0.84, 1.14)	
Triglycerides (mmol/l)				
<1.7	327	1769	0.90 (0.79, 1.02)	0.42
≥1.7	304	1802	0.98 (0.87, 1.11)	
Randomised BP lowering treatment				
BP treatment	296	1763	0.97 (0.86, 1.10)	0.63
Placebo	335	1813	0.90 (0.80, 1.02)	
Randomised intensive blood glucose control				
Intensive	309	1766	0.96 (0.85, 1.09)	0.77
Standard	322	1477	0.91 (0.80, 1.03)	

*Adjusted for age, sex, region, randomised treatment, history of macrovascular disease, duration of diabetes, current smoking status, systolic BP, BMI, urinary albumin/creatinine ratio, eGFR, HbA_{1c}, HDL-cholesterol, triacylglycerols, and use of aspirin or other antiplatelet agents, statins or other lipid-lowering agents, β-blockers, and ACE inhibitors or angiotensin receptor blockers
